# Association between *Mycoplasma pneumoniae* infection and adverse pregnancy outcome: a propensity score weighting study

**DOI:** 10.3389/fcimb.2025.1663272

**Published:** 2025-12-24

**Authors:** Caihua Yang, Haoxuan Jiang, Linyan Li, Ping Zheng, Yilei Li, Ying Wu

**Affiliations:** 1 Clinical Pharmacy Center, Nanfang Hospital, Southern Medical University, Guangzhou, China; 2 School of Biomedical Engineering, Hainan University, Sanya, China; 3 Department of Biostatistics, School of Public Health, Southern Medical University, Guangzhou, China; 4 Hainan Lecheng Institute of Real World Study, The Administration of Boao Lecheng International Medical Tourism Pilot Zone, Hainan, Qionghai, China

**Keywords:** advanced maternal age, COVID-19, *Mycoplasma pneumoniae*, pregnancy, propensity score

## Abstract

**Introduction:**

After COVID-19 pandemic, there has been an upward trend in *Mycoplasma pneumoniae* (*M. pneumoniae*) infections across Asia. The COVID-19-induced immunological impairment may increase the risk of adverse outcomes in *M. pneumoniae*-infected patients, yet studies in this area remain limited. We investigated the association between *M. pneumoniae* infection and adverse pregnancy outcomes in the post-COVID-19 era.

**Methods:**

We conducted a single-center cohort study in Guangzhou, China, from February 2023 to June 2024, involving pregnant women. A total of 186 participants were included, with 49 in the *M. pneumoniae* group (tested positive for *M. pneumoniae* immunoglobulin M antibody (MP IgM)) and 137 in the control group. Propensity score weighting analysis was performed to control bias and estimate the effect size.

**Results:**

The incidence of adverse pregnancy outcomes in the *M. pneumoniae* group was not significantly different from that in the control group. The odds ratio (OR) for adverse maternal events after propensity score weighting (PSW) was 1.25 (95% confidence interval [CI], 0.62 to 2.55; *p* = 0.530), and the PSW OR for adverse neonatal events was 0.95 (95% CI, 0.49 to 1.84; *p* = 0.884). However, in the subgroups of advanced maternal age (AMA, age ≥ 35, n=29) and primiparous women (n=80), the incidence of adverse pregnancy outcomes was significantly higher in the *M. pneumoniae* group. Additionally, the clinical manifestations of *M. pneumoniae* infection in the post-COVID-19 era were consistent with those observed prior to the pandemic.

**Conclusions:**

In the post-COVID-19 era, evidence remains insufficient to conclude that *M. pneumoniae* infection increases the risk of adverse pregnancy outcomes in the general pregnant population. Exploratory subgroup analyses suggest possible signals of risk within subgroups of AMA and primiparous women.

## Introduction

During the COVID-19 pandemic, the prevention and control measures, or non-pharmaceutical interventions (NPIs), blocked the transmission of many respiratory pathogens, including *M. pneumoniae* (Meyer Sauteur et al., 2022). Therefore, during the period of NPIs, the detection rate of *M. pneumoniae* was extremely low, until the first half of 2023, when there was an observable resurgence, indicating a new outbreak of *M. pneumoniae*, particularly in Asia (Meyer Sauteur et al., 2024).

It is estimated that 97% of the population in China had been infected with SARS-CoV-2 before January 2023 (Goldberg et al., 2023). Growing evidence indicates that SARS-CoV-2 infection can induce a prolonged phase of immune suppression and inflammatory injury, characterized by reduced counts of natural killer (NK) cells, lymphocytes, and monocytes, alongside elevated levels of inflammatory cytokines (Peluso et al., 2021; Ryan et al., 2022). Notably, immune dysregulation and inflammatory damage are recognized as key pathogenic mechanisms in severe *M. pneumoniae* pneumonia (SMPP) (Zhang et al., 2024; Yang et al., 2024). This suggests that the COVID-19-induced immunological impairment may potentially increase the risk of *M. pneumoniae*-infected patients progressing to severe disease. Patients with coinfection (COVID-19 and *M. pneumoniae*) have higher mortality compared with patients with just COVID-19 disease (Amin et al., 2021). This raises our interest in whether the prognosis of *M. pneumoniae* infection would become worse in the post-COVID-19 era (the period after January 2023 (Li et al., 2024)).

Pneumonia in pregnancy also results in low-birth-weight neonates in 33.9% of cases compared with 13.6% of controls (Goodnight and Soper, 2005). Tang et al. observed high incidences of adverse fetal outcomes in patients with severe pneumonia (Tang et al., 2018). Similarly, Chen et al. found that women with pneumonia during pregnancy had significantly higher risk of low birth weight, preterm birth, small for gestational age (SGA), low Apgar scores, cesarean section (CS), and preeclampsia/eclampsia, compared to without pneumonia (Chen et al., 2012). In the pregnant patient, pneumonia is the most frequent cause of fatal non-obstetric infection and *M. pneumoniae* is a common organism identified (Lim et al., 2001). From a biological perspective, a prior SARS-CoV-2 infection could potentially modify the maternal immune response to a subsequent *M. pneumoniae* infection, possibly leading to a more pronounced inflammatory response or an altered clinical course. Such a shift in host-pathogen interaction might result in maternal-fetal outcomes that differ from those observed in the pre-pandemic era, when the immune system had not been primed by SARS-CoV-2. Nevertheless, it remains unclear whether infection with *M. pneumoniae* affects adverse pregnancy outcomes in pregnant women in the post-COVID-19 era.

This paper aims to investigate the association between infection with *M. pneumoniae* and adverse pregnancy outcomes in pregnant women in the post-COVID-19 era, as well as to report the clinical manifestations of pneumonia caused by *M. pneumoniae.*

## Methods

### Study design and population

**Figure 1:**
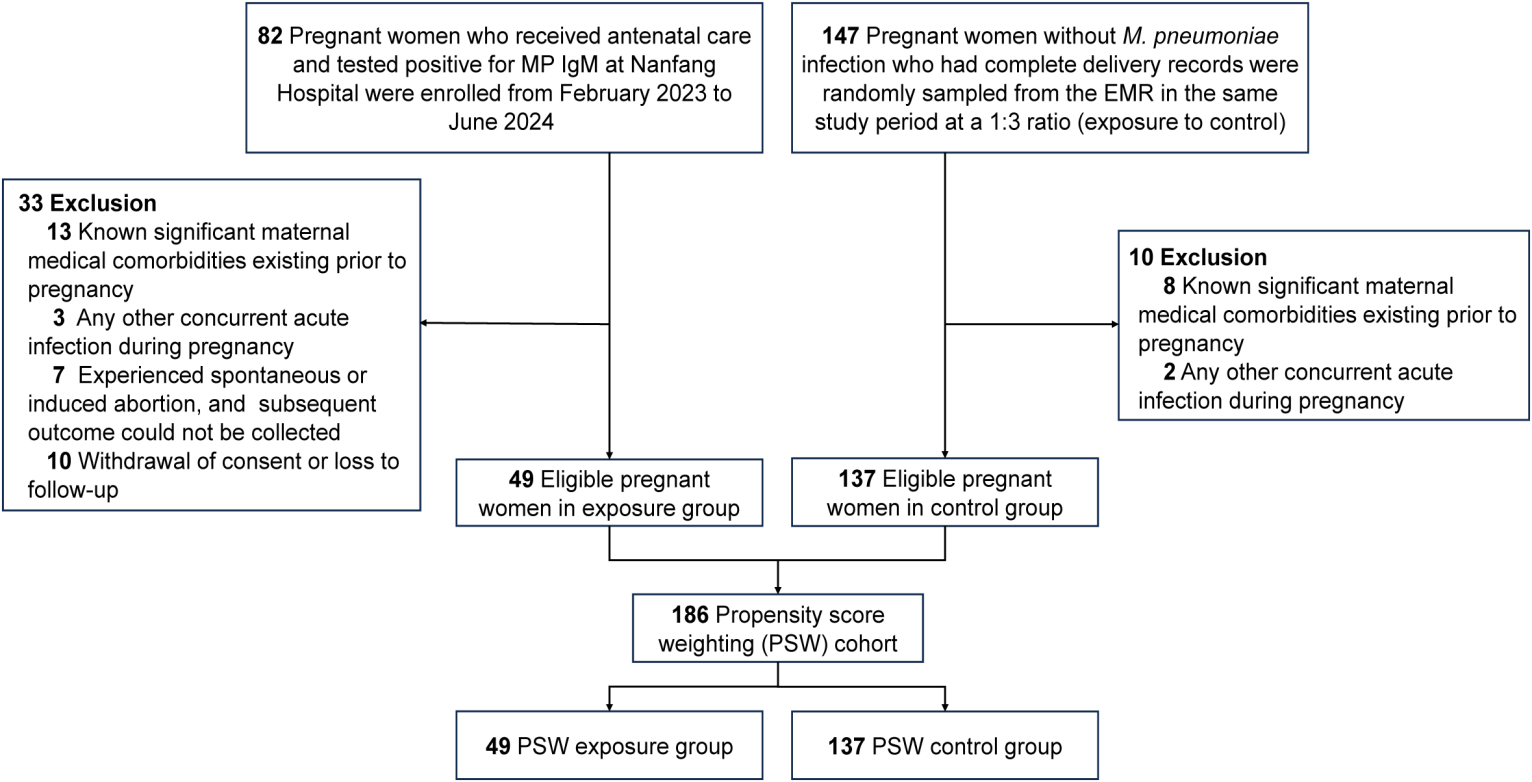
Selection flow chart. EMR, electronic medical record; MP IgM, *M. pneumoniae* immunoglobulin M antibody; PSW, Propensity Score Weighting.

This study, conducted at Nanfang Hospital (Guangzhou, China) between February 1, 2023 and June 21, 2024, utilized a prospectively collected cohort for the exposure group combined with a retrospectively selected control group. The inclusion criteria were as follows: (1) Pregnant women receiving antenatal care at the study site during the study period. (2) Provision of informed consent to participate in the study. The exclusion criteria were: (1) Known significant maternal medical comorbidities existing prior to pregnancy, including but not limited to autoimmune diseases, severe cardiac or renal dysfunction, and poorly controlled diabetes and hypertension. (2) The presence of any other concurrent acute infection during pregnancy. (3) Patients who experienced spontaneous or induced abortion, from whom subsequent outcome data could not be collected. (4) Withdrawal of consent or loss to follow-up during the study period. Patients who met the inclusion criteria and tested positive for *M. pneumoniae* immunoglobulin M antibody (MP IgM) during pregnancy were enrolled as the exposure group. Data for the exposure group were prospectively collected. Controls were retrospectively identified as patients without *M. pneumoniae* infection who met the inclusion criteria during the same study period. Given a 1:3 exposure-to-control ratio, participants with complete delivery records were randomly sampled from the electronic medical record (EMR) system. The complete patient selection flow chart is shown in **Figure 1**. Finally, 186 pregnant women remained in the final analysis, with 49 in the *M. pneumoniae* group and 137 in the control group. This study was approved by the Medical Ethics Committee of Nanfang Hospital affiliated to Southern Medical University (ID: NFEC- 2023-116).

### Data collection and measurement

For the exposure group, demographic and baseline characteristics were collected from the participants at the time of recruitment (i.e., at the diagnosis of infection). Among them, 77.6% (n=38) of participants were infected from 28 weeks of pregnancy to delivery, while 22.4% (n=11) were infected between 0 and 28 weeks of pregnancy. Participants were prospectively followed up from recruitment until delivery. Clinical manifestations of *M. pneumoniae* infection were recorded as they developed during follow-up, and final adverse pregnancy outcomes were collected at the time of delivery. For the control group, demographics, baseline characteristics, and adverse pregnancy outcomes were systematically extracted from the EMR. Demographic and baseline characteristics included age, BMI, gravidity, history of abortion (encompassing both spontaneous and induced abortions), and history of chronic diseases.

Adverse pregnancy outcomes were categorized into maternal and neonatal outcomes. Adverse maternal outcomes included six variables: CS, postpartum hemorrhage (PPH), polyhydramnios or oligohydramnios, amniotic fluid contamination, placental abruption, and premature rupture of membranes (PROM). Adverse neonatal outcomes included eight variables: fetal heart rate variability (HRV), preterm birth, fetal distress, other neonatal infection, neonatal length, neonatal weight, neonatal head circumference, and the one-minute Apgar score. To increase the number of outcome events, the primary adverse maternal outcome in this study was defined as adverse maternal events, which is a composite outcome, encompassing CS, PPH, polyhydramnios or oligohydramnios, amniotic fluid contamination, placental abruption, and PROM. Similarly, the primary adverse neonatal outcome was defined as adverse neonatal events, including fetal HRV, preterm birth, birth asphyxia (defined as a one-minute Apgar score ≤ 7), low birth weight (defined as neonatal weight< 2.5 kg), fetal distress, and other neonatal infections. Each individual variable was considered a secondary outcome. The encoding of each variable is presented in **Supplementary Table S1**.

### Statistical analysis

Descriptive analyses were performed for all the demographic and baseline characteristics. Continuous variables were expressed as the means with standard deviations. And categorical variables were expressed as counts and percentages. Missing values in demographic and baseline characteristics were imputed via multiple imputation (5 imputation datasets) (Harel and Zhou, 2007). Continuous variables were imputed using predictive mean matching, while categorical variables had no missing value. The proportion of missing data for each variable in each group are reported in **Supplementary Table S1**.

Propensity scores (Austin, 2011), which represent the probability of infection with *M. pneumoniae*, were estimated with a multivariable logistic regression model incorporating all demographics and baseline characteristics. Specifically, age and BMI were included as continuous variables, history of abortion and history of chronic diseases were treated as binary variables, while gravidity was treated as a three-level categorical variable. Stabilized propensity score weighting (PSW) based on propensity scores was used to balance the baseline characteristics between the *M. pneumoniae* group and the control group (Austin and Stuart, 2015). The balance of all baseline characteristics was evaluated via the absolute standardized mean difference (ASMD) in both unadjusted data and PSW data (**Table 1**). An ASMD of< 0.10 was defined as an acceptable balance (Austin, 2009). After applying PSW, weighted generalized linear models were employed to compare the difference of multiple adverse pregnancy outcomes between the *M. pneumoniae* and control groups (identity link function for continuous outcome variables and probit link function for binary outcomes). For continuous outcome variables, mean differences were used to quantify the exposure effect, whereas for binary outcomes, odds ratios (OR) were applied to measure the effect. Propensity score matching (PSM) was performed as a sensitivity analysis due to the loss of sample size.

**Table 1:** Baseline characteristics of participants before and after propensity score weighting.

Baseline	Unadjusted			PSW		
	Control (n=137)	*M. pneumoniae* (n=49)	ASMD	Control (n=137)	*M. pneumoniae* (n=49)	ASMD
Age, Mean ± SD	30.42 ± 4.37	29.18 ± 3.79	0.301	30.04 ± 4.37	29.65 ± 3.62	0.098
BMI, Mean ± SD	26.5 ± 3.74	25.65 ± 3.51	0.238	26.28 ± 3.67	26.12 ± 3.62	0.044
Gravidity, n (%)			0.198			0.027
1	58 (42.34)	22 (44.90)		58 (42.45)	20 (41.80)	
2	46 (33.58)	19 (38.78)		48 (35.28)	18 (36.56)	
3	33 (24.09)	8 (16.33)		31 (22.27)	11 (21.64)	
History of abortion, n (%)	40 (29.20)	15 (30.61)	0.031	41 (30.35)	16 (33.55)	0.069
History of chronic diseases, n (%)	22 (16.06)	17 (34.69)	0.438	29 (21.29)	10 (21.34)	0.001

An ASMD of< 0.10 was defined as an acceptable covariate balance. ASMD, absolute standardized mean difference; BMI, body mass index; PSW, propensity score weighting; SD, standard deviation.

Firth’s penalized logistic regression (Chaudhry et al., 2025) was used to correct for small sample bias (also known as sparse data bias (Greenland et al., 2016)) due to limited sample size and complete separation (Gosho et al., 2023).

We also performed subgroup analyses to investigate the homogeneity of *M. pneumoniae* infection for adverse pregnancy outcomes across clinically important subgroups (age, gravidity, history of abortion, history of chronic diseases). In addition, subgroup analysis can help identify potential high-risk populations, providing guidance for disease prevention and treatment. The two-tailed Wald test was used to assess the significance of regression coefficients, and statistical significance was defined as a *p* < 0.05. All the statistical analyses were conducted via R software (version 4.3.1) and a detailed list of all R packages and versions used in this study is provided in **Supplementary Table S2**.

## Results

### Demographic and baseline characteristics

The demographics and baseline characteristics of patients with exposure and control groups were compared in unadjusted and PSW-adjusted data (**Table 1**). In the unadjusted data, significant disparities were noted in the demographic and baseline variables between the two groups. These differences were markedly reduced after PSW, with the ASMD decreasing significantly, indicating a successful balance of demographic and baseline variables (all ASMDs ≤ 0.10). For example, the age was comparable between the exposure (29.65) and control (30.04) groups after PSW. The BMI, gravidity, the history of abortion and chronic diseases also showed small differences after PSW. The characteristics after PSM were displayed in **Supplementary Table S3**.

### Clinical manifestations and laboratory findings of infection with *M. pneumoniae*

We analyzed the clinical manifestations and laboratory test results of 49 pregnant women in the *M. pneumoniae* infection group (**Table 2**). The clinical manifestations of *M. pneumoniae* infection were predominantly upper respiratory tract symptoms, including fever in 33 patients (67.35%), cough in 34 patients (69.39%), expectoration in 20 patients (40.82%), pharyngodynia in 20 patients (40.82%), nasal congestion in 13 patients (26.53%), and rhinorrhea in 11 patients (22.45%). In addition, systemic toxic symptoms were reported, including headache (16.33%) and asthenia (12.24%). Furthermore, three patients (6.12%) exhibited dyspnea.

**Table 2:** Clinical manifestations and laboratory test results of infection with *M. pneumoniae*.

Characteristics	M. pneumoniae group(n=49)
Fever, n (%)	33 (67.35)
Cough, n (%)	34 (69.39)
Expectoration, n (%)	20 (40.82)
Pharyngodynia, n (%)	20 (40.82)
Nasal congestion, n (%)	13 (26.53)
Rhinorrhea, n (%)	11 (22.45)
Headache, n (%)	8 (16.33)
Asthenia, n (%)	6 (12.24)
Dyspnea, n (%)	3 (6.12)
Number of symptoms	3.29 ± 1.38
Duration of symptoms, days	8.63 ± 8.62
Duration of hospitalization, days	7.41 ± 10.16
Any complication of *M. pneumoniae* infection, n (%)	2 (4.1)
WBC, $1\times 10^{9}/L$	9.35 ± 2.77
LYM, $1\times 10^{9}/L$	1.09 ± 0.64
PLT, $1\times 10^{9}/L$	237.71 ± 62.93
Hb, $1\times 10^{9}/L$	111.65 ± 10.41
CRP, mg/L	26.76 ± 26.79
PCT, ng/mol	2.10 ± 12.91
IL-6, pg/mL	35.63 ± 58.29
ALT, U/L	16.00 ± 14.75
AST, U/L	21.04 ± 8.34
SCr, μmol/L	47.02 ± 10.54

77.6% (n=38) of participants were infected from 28 weeks of pregnancy to delivery, while 22.4% (n=11) were infected between 0 and 28 weeks of pregnancy. Values are presented as number (%) and mean ± SD. ALT, alanine aminotransferase; AST, aspartate transaminase; CRP, C-reactive protein; Hb, hemoglobin; IL-6, Interleukin-6; LYM, lymphocyte; PCT, procalcitonin; PLT, platelets; SCr, serum creatinine; SD, standard deviation; WBC, white blood cell.

Laboratory test results encompassed hematological parameters, inflammatory biomarkers, and hepatorenal function profiles. Hematological analysis revealed white blood cell (WBC, $9.35\times 10^{9}/L$) and platelet (PLT, $237.71\times 10^{9}/L$) counts within normal reference ranges. In contrast, lymphocyte (LYM, $1.09\times 10^{9}/L$) and hemoglobin (Hb, $111.65\;\times 10^{9}/L$) levels demonstrated mild decreases compared to normal threshold. As expected, all inflammatory markers in the *M. pneumoniae* group showed significantly elevated average values, including C-reactive protein (CRP, 26.76 mg/L), procalcitonin (PCT, 2.10 mg/L), and interleukin-6 (IL-6, 35.63 mg/L). Hepatorenal function indices, however, remained within clinically normal limits, with alanine aminotransferase (ALT, 16.00 U/L, aspartate aminotransferase (AST, 21.04 U/L), and serum creatinine (SCr, 47.02 μmol/L).

### The association of *M. pneumoniae* infection and adverse pregnancy outcome

The primary adverse maternal outcome is adverse maternal events. As shown in **Table 3**, in the unadjusted analysis, a total of 126 pregnant women experienced adverse maternal events, with 49 cases (69.39%) in the *M. pneumoniae* group and 92 cases (67.15%) in the control group. The PSW-adjusted OR was 1.25 (95% CI, 0.62 to 2.55; *p* = 0.530), suggesting no significant difference between *M. pneumoniae* infection and non-infection. As for the secondary outcomes, our research revealed that CS, PPH, amniotic fluid contamination, and PROM were almost identical between the control group and *M. pneumoniae* group, while polyhydramnios or oligohydramnios and placental abruption showed considerable differences between the control group and *M. pneumoniae* group, but all of these outcomes showed no statistically significant differences both for unadjusted analysis and PSW analysis.

**Table 3:** Effects of *M. pneumoniae* infection on pregnant women before and after propensity score weighting.

Outcomes	Control (n=137)	*M. pneumoniae* (n=49)	Unadjusted		PSW	
			OR/MD	*p*	OR/MD	*p*
Adverse maternal events, n (%) #	92 (67.15)	34 (69.39)	1.11 (0.55-2.25)	0.774	1.25 (0.62-2.55)	0.530
CS, n (%)	60 (43.80)	28 (57.14)	1.71 (0.88-3.32)	0.112	1.83 (0.94-3.55)	0.075
PPH, n (%) ^†^	1 (0.73)	0 (0.00)	0.92 (0.04-24.09)	0.959	0.91 (0.03-23.74)	0.954
Polyhydramnios or oligohydramnios, n (%)	16 (11.68)	11 (22.45)	2.19 (0.93-5.15)	0.072	2.19 (0.93-5.17)	0.074
Amniotic fluid contamination, n (%)	16 (11.68)	6 (12.24)	1.06 (0.39-2.89)	0.916	1.52 (0.59-3.92)	0.388
Placental abruption, n (%)	1 (0.73)	1 (2.04)	2.83 (0.17-47.05)	0.466	7.16 (0.62-82.09)	0.113
PROM, n (%)	34 (24.82)	10 (20.41)	0.78 (0.35-1.73)	0.535	0.64 (0.28-1.49)	0.302
Adverse neonatal events, n (%) #	65 (47.45)	24 (48.98)	1.06 (0.55-2.05)	0.854	0.95 (0.49-1.84)	0.884
Fetal HRV, n (%)	26 (18.98)	14 (28.57)	1.71 (0.80-3.64)	0.165	1.44 (0.67-3.11)	0.347
Preterm infant, n (%)	17 (12.41)	8 (16.33)	1.38 (0.55-3.45)	0.492	1.34 (0.53-3.38)	0.532
Fetal distress, n (%)	24 (17.52)	10 (20.41)	1.21 (0.53-2.76)	0.654	1.81 (0.84-3.93)	0.131
Other neonatal infection, n (%)	7 (5.11)	6 (12.24)	2.59 (0.82-8.19)	0.104	2.65 (0.85-8.33)	0.094
Neonatal length, Mean ± SD	49.58 ± 3.01	49.31 ± 2.22	-0.27 (-1.20-0.66)	0.566	-0.13 (-1.04-0.79)	0.785
Neonatal weight, Mean ± SD	3.05 ± 0.53	3.01 ± 0.52	-0.05 (-0.22-0.12)	0.586	0.02 (-0.15-0.19)	0.845
Neonatal head circumference, Mean ± SD	33.04 ± 1.93	32.96 ± 1.55	-0.08 (-0.68-0.53)	0.800	0.09 (-0.50-0.69)	0.755
One-minute Apgar score, Mean ± SD	8.93 ± 0.30	8.90 ± 0.51	-0.04 (-0.16-0.08)	0.554	-0.05 (-0.17-0.08)	0.464

#The primary outcome. ^†^Firth’s penalized logistic regression to correct sparse data bias.

CS, cesarean section; HRV, heart rate variability; PSW, propensity score weighting; MD, mean difference; OR, odds ratio; PPH, postpartum hemorrhage; PROM, premature rupture of membranes; SD, standard deviation.

Additionally, a total of 186 neonates were delivered. In the PSW analysis, a total of 89 neonates experienced adverse events, with 24 cases (48.98%) in the *M. pneumoniae* group and 65 cases (47.45%) in the control group. The PSW OR between the two groups was 0.95 (95% CI, 0.49 to 1.84; *p* = 0.884), suggesting no significant difference between groups. The PSW and unadjusted analyses yielded consistent results, with an unadjusted OR of 1.06 (48.98% vs. 47.45%; 95% CI, 0.55 to 2.05; *p* = 0.854). Similarly, for secondary adverse neonatal outcomes, including fetal HRV, preterm birth, fetal distress, other neonatal infections, neonatal length, neonatal weight, neonatal head circumference, and the one-minute Apgar score, there was no significant difference between groups. Similar patterns were found in the PSM analysis (**Supplementary Table S4**).

### Subgroup analysis

**Figure 2:**
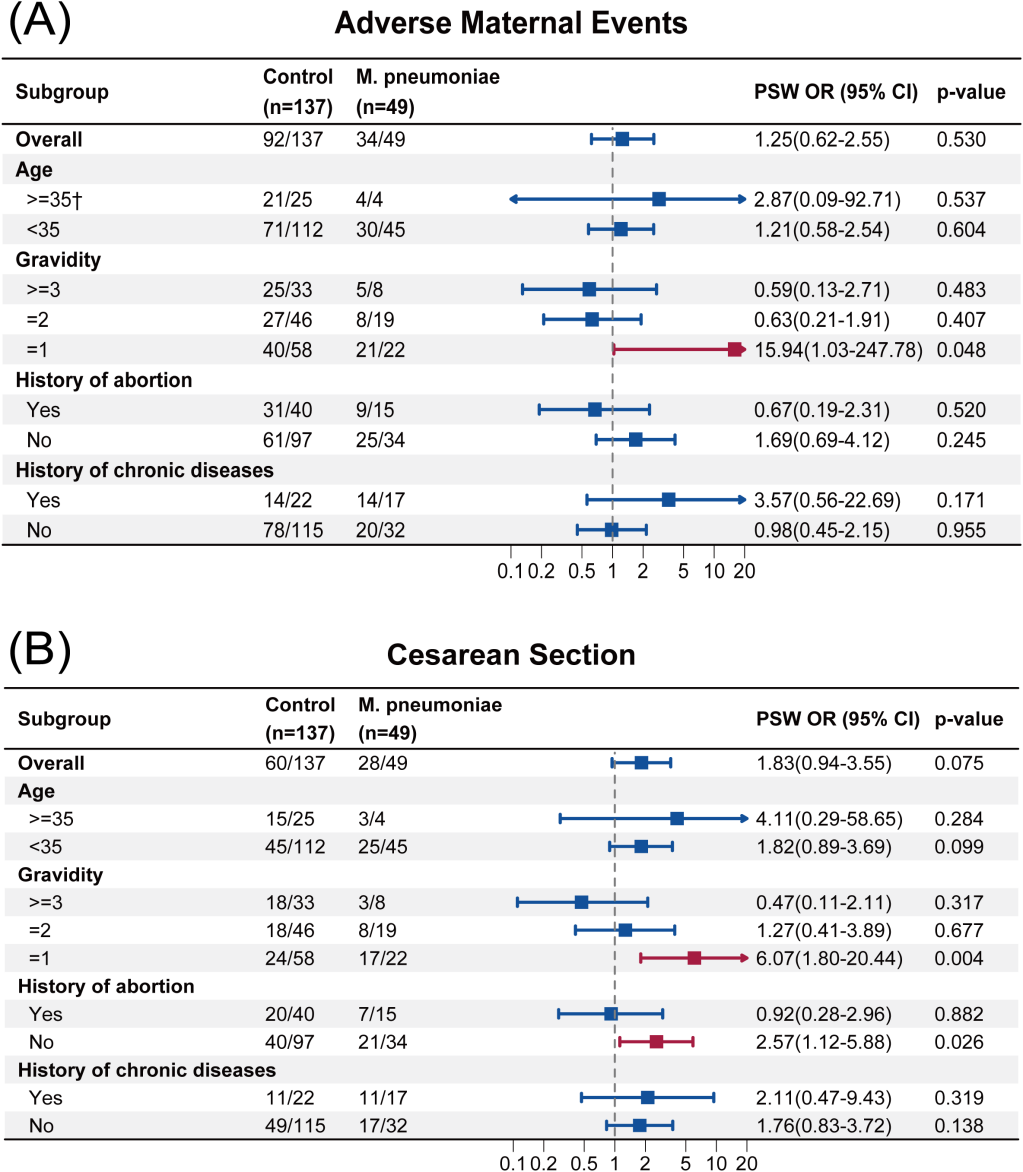
**(A)** Subgroup analysis of adverse maternal events. **(B)** Subgroup analysis of cesarean section. ^†^Firth’s penalized logistic regression to correct sparse data bias. PSW, propensity score weighting; OR, odds ratio.

**Figure 3:**
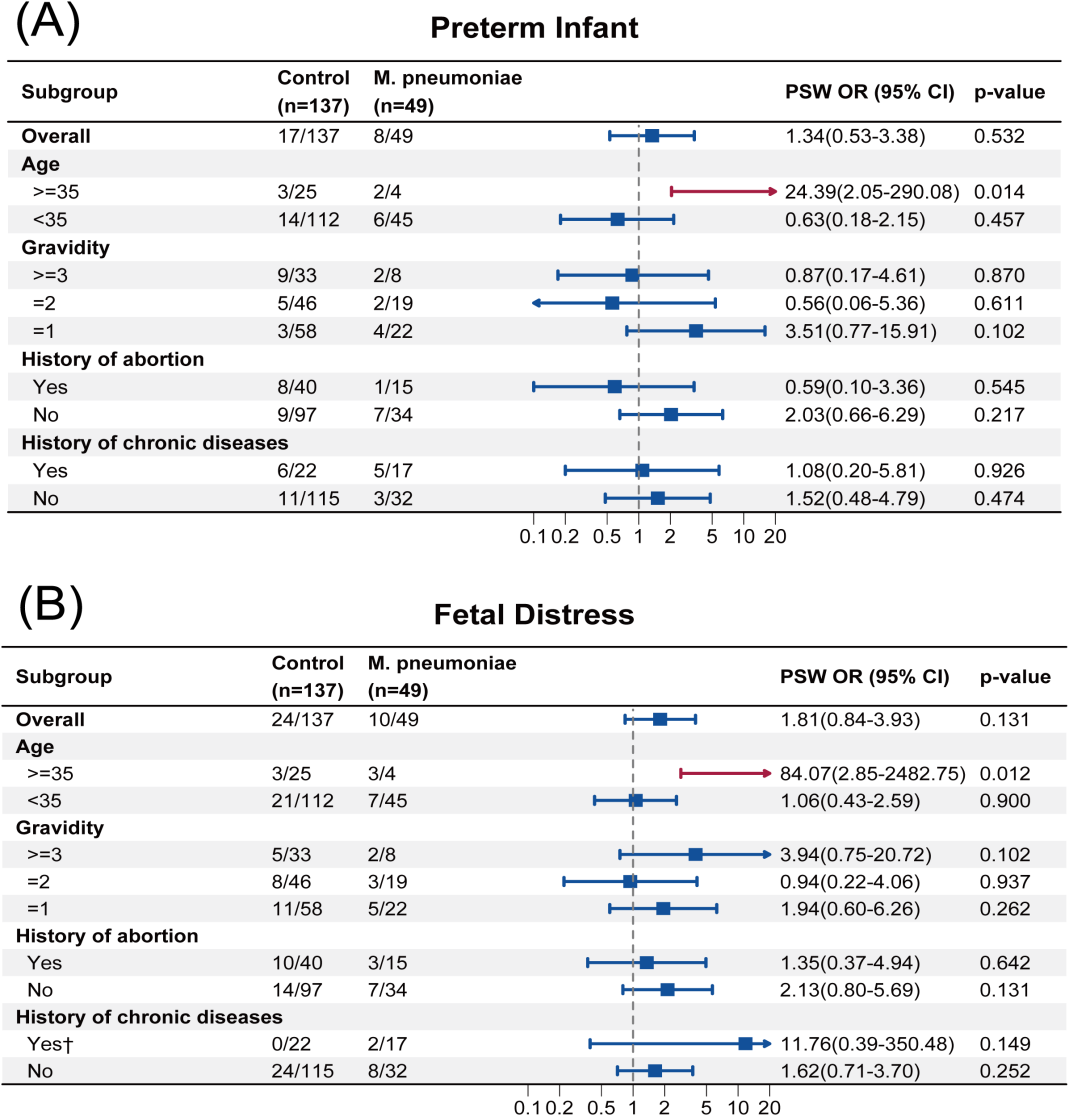
**(A)** Subgroup analysis of preterm infant. **(B)** Subgroup analysis of fetal distress.

The result of subgroup analysis was presented in **Figures 2**, **3**. Firth’s penalized logistic regression was also applied to subgroup analyses. In primiparous women (Gravidity=1), *M. pneumoniae* infection was associated with increased odds of adverse maternal events (PSW OR, 15.94; 95% CI, 1.03 to 247.78; *p* = 0.048). With regard to CS, we also found that for primiparous women, infection with *M. pneumoniae* increased the risk of undergoing a CS (PSW OR, 6.07; 95% CI, 1.80 to 20.44; *p* = 0.004). A similar result was observed among participants without history of abortion, with an PSW OR of 2.57 (95% CI, 1.12 to 5.88; *p* = 0.026).

The PSW OR for most adverse neonatal outcomes among AMA was statistically significant. For example, the PSW OR of preterm infant was 24.39 (95% CI, 2.05 to 290.08; *p* = 0.014), and that of fetal distress was 84.07 (95% CI, 2.85 to 2482.75; *p* = 0.012), which indicated that, among AMA, infection with *M. pneumoniae* increased the risk of preterm birth and fetal distress. The subgroup analyses based on PSW for the other adverse pregnancy outcomes are presented in **Supplementary Figure S1**–**S12**. Notably, statistical significance was observed in 6 out of 9 adverse neonatal outcomes among the AMA subgroup (**Figure 3**, **Supplementary Figure S6**-**S12**), suggesting possible signals of risk associated with *M. pneumoniae* infection in AMA.

## Discussion

The COVID-19 pandemic has significantly diminished the populace’s immunity (Wu et al., 2024), which may result in a poorer prognosis when pneumonia occurs post-COVID-19 era compared to pre-COVID-19 era. This study focused on the outbreak of *M. pneumoniae* infections following the COVID-19 pandemic and provided some valuable insights into the potential impact of *M. pneumoniae* infection on adverse pregnancy outcomes among pregnant women, based on data from Guangzhou, China.

We first analyzed the clinical manifestations and laboratory findings of hospitalized pregnant women with *M. pneumoniae* infection after the COVID-19 pandemic (2023). Upper respiratory tract infection (URI) was the most common clinical presentation of *M. pneumoniae* infection, with the majority of pregnant patients exhibiting fever and cough as predominant symptoms (Layani-Milon et al., 1999). Respiratory manifestations were the most prominent, often accompanied by pronounced pharyngodynia, nasal congestion, headache, and asthenia, indicative of systemic toxicity. In contrast, extrapulmonary manifestations were uncommon and generally mild. These findings were consistent with the Expert Consensus on the Diagnosis and Treatment of *M. pneumoniae* Pneumonia in Adults, developed by the Infection Group of the Respiratory Society of the Chinese Medical Association. These clinical features were also comparable to those reported in adult *M. pneumoniae* infections both before and after the COVID-19 pandemic (Centers for Disease Control and Prevention (CDC), 2013; Li et al., 2024). Laboratory findings revealed that pregnant women with *M. pneumoniae* infection had white blood cell (WBC) counts approaching the upper limit of the normal adult range, while lymphocyte counts were slightly below the normal threshold, aligning with previous reports (Li et al., 2024). *M. pneumoniae* infection has been associated with a broad decline in immune cell populations, including lymphocytes, CD3^+^ T cells, CD4^+^ T cells, CD8^+^ T cells, and B cells, suggesting potential immunological alterations. Regarding inflammatory markers, levels of C-reactive protein (CRP), procalcitonin (PCT), and interleukin-6 (IL-6) were significantly elevated, indicating a robust inflammatory response. This trend is consistent with the findings of Li et al (Li et al., 2024), who reported similar elevations in CRP, PCT, and IL-6 in pediatric *M. pneumoniae* infections in Guangzhou following the COVID-19 pandemic, with these markers correlating with infection severity. Moreover, our results demonstrated no significant impairment of hepatic or renal function in pregnant women with *M. pneumoniae* infection. However, the potential impact of prolonged or severe *M. pneumoniae* infection on hepatic and renal function requires further investigation.

Following propensity score analysis, our findings suggest that in the post-COVID-19 era, evidence remains insufficient to conclude that *M. pneumoniae* infection increases the risk of adverse pregnancy outcomes in the overall population. However, some subgroups showed statistical significance. This contrast may be attributed to the study population being insufficiently specific to capture potential high-risk groups (e.g., the AMA population), thereby limiting our ability to detect statistical significance.

A possible reason for the lack of observed significance in the overall population is the small sample size of the cohort, with only 49 participants in the exposure group, which may have limited our ability to detect the differences (Sullivan and Feinn, 2012). Therefore, a *post-hoc* power analysis was conducted to evaluate the statistical power. The results showed that for the logistic regression analysis, a total sample of 186 participants (49 exposure group vs. 137 control group) achieves 54% power (implying a 46% Type II error rate) at a two-tailed 0.05 significance level to detect an OR of 2.00, assuming a baseline risk of 0.4 (**Supplementary Figure S13****).** A problem accompanying the small sample size was the low number of events (e.g., no participants in the *M. pneumoniae* group experienced postpartum hemorrhage), which can lead to considerable upward or downward bias in estimates obtained from standard ML. Given this unavoidable limitation, Firth’s penalized logistic regression was used to correct small sample bias, thereby preventing more extreme and implausible estimates. This issue of small sample size was particularly pronounced in the subgroup analyses. For instance, the AMA subgroup included only 29 participants.

Subgroup analyses for adverse maternal outcomes revealed two findings: *M. pneumoniae* infection significantly increased the risk of adverse maternal events and CS in primiparous women, while women without a history of abortion also exhibited a higher CS rate. These findings would be of substantial importance if the associations are confirmed in adequately powered studies. Prior to the COVID-19 pandemic, multiple clinical studies on pneumonia in pregnant women identified CS as a major adverse pregnancy outcome (Tang et al., 2018; Chen et al., 2012; Romanyuk et al., 2011). Our findings in the post-pandemic era are consistent with these reports, particularly in the subgroup of pregnant women with infection with *M. pneumoniae*. This increased CS rate may be attributed to the elevation of inflammatory cytokines following pathogen infection, which can impair uterine contractility and cervical dilation, subsequently increasing the likelihood of CS. Studies have demonstrated that the activation of inflammatory transcription factors such as NF-κB may directly upregulate genes associated with uterine contractions, thereby promoting the contractility of uterine smooth muscle (Mendelson, 2009). Furthermore, monocytes recruited to the cervix and myometrium, upon activation, release pro-inflammatory cytokines including IL-1β, TNF-α, and IL-6, which participate in cervical ripening, membrane rupture, and the initiation of labor (Obeagu, 2025). *M. pneumoniae* infection has been shown to significantly elevate the levels of cytokines such as IL-6, IL-8, and TNF-α (Zhao et al., 2019). The findings from Li et al (Li et al., 2024). indirectly lend support to the potential importance of this mechanism. Notably, within the amniotic fluid environment, IL-6 and IL-8 levels have been established as robust predictors of adverse fetal outcomes (McCartney et al., 2021). The impact of infection with *M. pneumoniae* on adverse maternal outcomes was more pronounced in primiparous women, potentially due to both psychological and physiological factors. Psychologically, the absence of prior childbirth experience may contribute to heightened fear and anxiety, which could affect stress responses and lead to labor abnormalities. Physiologically, the uterus and birth canal of primiparous women may exhibit increased susceptibility to infection, further predisposing them to adverse outcomes. Similarly, pregnant women without a history of abortion may have a relatively fragile reproductive system, lacking prior immunological adaptation to pregnancy-related inflammatory challenges, which could make them more vulnerable to the adverse effects of *M. pneumoniae*.

In terms of adverse neonatal outcomes, subgroup analyses revealed that, in AMA, the incidence of preterm birth, fetal distress, and other infections was significantly higher in the *M. pneumoniae* group. Moreover, neonates in this subgroup exhibited a marked tendency toward reduced birth length, weight, and head circumference. Although these findings from exploratory subgroup analyses require further validation given the limited sample size, several mechanisms may explain these observations. First, AMA is inherently associated with multiple obstetric risk factors, predisposing to adverse pregnancy outcomes, which is consistent with the meta-analysis findings of Lean et al (Lean et al., 2017a). A widely accepted explanation is that accelerated placental senescence in AMA pregnancies leads to altered nutrient transport and vascular function, thereby compromising the intrauterine environment for fetal growth. Similar characteristics have been observed in aged murine models (Lean et al., 2017b). Second, the genetic integrity of oocytes declines with maternal aging (Cimadomo et al., 2018), and while the relationship between this phenomenon and adverse pregnancy outcomes remains to be fully elucidated, it warrants further investigation. Additionally, in pregnancies at AMA complicated by infection with *M. pneumoniae*, the infection and its associated inflammatory response constitute a major risk factor for preterm birth (Cappelletti et al., 2016). This heightened inflammatory burden may impose additional stress on the placenta and uterus, increasing the risk of fetal distress and further restricting fetal growth, ultimately leading to reductions in neonatal birth length, weight, and head circumference. These findings align with clinical studies conducted before the COVID-19 pandemic on pneumonia-complicated pregnancies (Chen et al., 2012).

These findings should be interpreted in the context of specific limitations. First, the single-center design of this study may restrict the generalizability of our findings. Additionally, statistical power was insufficient, as *post-hoc* analysis indicated 54% power overall, implying a 46% Type II error rate. Similarly, the exploratory subgroup analyses were constrained by small sample sizes (particularly in the AMA subgroup), complete separation, and the potential for spurious findings due to multiple comparisons across outcomes and subgroups. Consequently, the subgroup findings are hypothesis-generating and require validation in larger, adequately powered studies.

## Conclusions

In the post-COVID-19 era, evidence remains insufficient to conclude that *M. pneumoniae* infection increases the risk of adverse pregnancy outcomes in the general pregnant population. However, our findings suggest potential risks specifically within subgroups of AMA and primiparous women. Despite limitations regarding sample size and the single-center design, it provides clinically relevant insights into patterns of *M. pneumoniae* infection in the post-COVID-19 era. These findings offer valuable guidance for clinical management and inform therapeutic decision-making following infection.

## Data Availability

The data and analytical code used in this study are available from the corresponding author upon reasonable request. Requests to access these datasets should be directed to CY, xiaoyang856@163.com.

## References

[B1] Amin D. McKitish K. Shah P. S. (2021). Association of mortality and recent Mycoplasma pneumoniae infection in COVID-19 patients. J. Med. Virol. 93, 1180–1183. doi: 10.1002/jmv.26467, PMID: 32852080 PMC7461379

[B2] Austin P. C. (2009). Using the standardized difference to compare the prevalence of a binary variable between two groups in observational research. Commun. Stat - Simulation Comput. 38, 1228–1234. doi: 10.1080/03610910902859574

[B3] Austin P. C. (2011). An introduction to propensity score methods for reducing the effects of confounding in observational studies. Multivariate Behav. Res. 46, 399–424. doi: 10.1080/00273171.2011.568786, PMID: 21818162 PMC3144483

[B4] Austin P. C. Stuart E. A. (2015). Moving towards best practice when using inverse probability of treatment weighting (IPTW) using the propensity score to estimate causal treatment effects in observational studies. Stat Med. 34, 3661–3679. doi: 10.1002/sim.6607, PMID: 26238958 PMC4626409

[B5] Cappelletti M. Della Bella S. Ferrazzi E. Mavilio D. Divanovic S. (2016). Inflammation and preterm birth. J. Leukoc. Biol. 99, 67–78. doi: 10.1189/jlb.3MR0615-272RR, PMID: 26538528

[B6] Centers for Disease Control and Prevention (CDC) (2013). Mycoplasma pneumoniae outbreak at a university - Georgia 2012. MMWR Morb Mortal Wkly Rep. 62, 603–606., PMID: 23903594 PMC4604853

[B7] Chaudhry S. A. Haj A. K. Ryu J. Jurgens S. J. Rodriguez Espada A. Wang X. . (2025). Population-scale studies of protein S abnormalities and thrombosis. JAMA 333, 1423. doi: 10.1001/jama.2025.0155, PMID: 40029645 PMC11877412

[B8] Chen Y.-H. Keller J. Wang I.-T. Lin C.-C. Lin H.-C. (2012). Pneumonia and pregnancy outcomes: a nationwide population-based study. Am. J. Obstet Gynecol 207, 288.e1–288.e7. doi: 10.1016/j.ajog.2012.08.023, PMID: 23021691 PMC7093888

[B9] Cimadomo D. Fabozzi G. Vaiarelli A. Ubaldi N. Ubaldi F. M. Rienzi L. (2018). Impact of maternal age on oocyte and embryo competence. Front. Endocrinol. (Lausanne) 9. doi: 10.3389/fendo.2018.00327, PMID: 30008696 PMC6033961

[B10] Goldberg E. E. Lin Q. Romero-Severson E. O. Ke R. (2023). Swift and extensive Omicron outbreak in China after sudden exit from “zero-COVID” policy. Nat. Commun. 14, 3888. doi: 10.1038/s41467-023-39638-4, PMID: 37393346 PMC10314942

[B11] Goodnight W. H. Soper D. E. (2005). Pneumonia in pregnancy. Crit. Care Med. 33, S390–S397. doi: 10.1097/01.ccm.0000182483.24836.66, PMID: 16215363

[B12] Gosho M. Ohigashi T. Nagashima K. Ito Y. Maruo K. (2023). Bias in odds ratios from logistic regression methods with sparse data sets. J. Epidemiol. 33, 265–275. doi: 10.2188/jea.JE20210089, PMID: 34565762 PMC10165217

[B13] Greenland S. Mansournia M. A. Altman D. G. (2016). Sparse data bias: a problem hiding in plain sight. BMJ 352, i1981. doi: 10.1136/bmj.i1981, PMID: 27121591

[B14] Harel O. Zhou X. (2007). Multiple imputation: review of theory, implementation and software. Stat Med. 26, 3057–3077. doi: 10.1002/sim.2787, PMID: 17256804

[B15] Layani-Milon M.-P. Gras I. Valette M. Luciani J. Stagnara J. Aymard M. . (1999). Incidence of Upper Respiratory TractMycoplasma pneumoniae Infections among Outpatients in Rhône-Alpes, France, during Five Successive Winter Periods. J. Clin. Microbiol. 37, 1721–1726. doi: 10.1128/jcm.37.6.1721-1726.1999, PMID: 10325314 PMC84933

[B16] Lean S. C. Derricott H. Jones R. L. Heazell A. E. P. (2017a). Advanced maternal age and adverse pregnancy outcomes: A systematic review and meta-analysis. PloS One 12, e0186287. doi: 10.1371/journal.pone.0186287, PMID: 29040334 PMC5645107

[B17] Lean S. C. Heazell A. E. P. Dilworth M. R. Mills T. A. Jones R. L. (2017b). Placental dysfunction underlies increased risk of fetal growth restriction and stillbirth in advanced maternal age women. Sci. Rep. 7, 9677. doi: 10.1038/s41598-017-09814-w, PMID: 28852057 PMC5574918

[B18] Li Y. Wu M. Liang Y. Yang Y. Guo W. Deng Y. . (2024). Mycoplasma pneumoniae infection outbreak in Guangzhou, China after COVID-19 pandemic. Virol. J. 21, 183. doi: 10.1186/s12985-024-02458-z, PMID: 39129001 PMC11318190

[B19] Lim W. S. Macfarlane J. T. Colthorpe C. L. (2001). Pneumonia and pregnancy. Thorax 56, 398–405. doi: 10.1136/thorax.56.5.398, PMID: 11312410 PMC1746055

[B20] McCartney S. A. Kapur R. Liggitt H. D. Baldessari A. Coleman M. Orvis A. . (2021). Amniotic fluid interleukin 6 and interleukin 8 are superior predictors of fetal lung injury compared with maternal or fetal plasma cytokines or placental histopathology in a nonhuman primate model. Am. J. Obstetrics Gynecology 225, 89.e1–89.e16. doi: 10.1016/j.ajog.2020.12.1214, PMID: 33412130 PMC8254735

[B21] Mendelson C. R. (2009). Minireview: fetal-maternal hormonal signaling in pregnancy and labor. Mol. Endocrinol. 23, 947–954. doi: 10.1210/me.2009-0016, PMID: 19282364 PMC2703595

[B22] Meyer Sauteur P. M. Beeton M. L. European Society of Clinical Microbiology and Infectious Diseases (ESCMID) Study Group for Mycoplasma and Chlamydia Infections (ESGMAC) ESGMAC Mycoplasma pneumoniae Surveillance (MAPS) study group (2024). Mycoplasma pneumoniae: delayed re-emergence after COVID-19 pandemic restrictions. Lancet Microbe 5, e100–e101. doi: 10.1016/S2666-5247(23)00344-0, PMID: 38008103

[B23] Meyer Sauteur P. M. Beeton M. L. Uldum S. A. Bossuyt N. Vermeulen M. Loens K. . (2022). Mycoplasma pneumoniae detections before and during the COVID-19 pandemic: results of a global survey 2017 to 2021. Euro Surveill 27, 2100746. doi: 10.2807/1560-7917.ES.2022.27.19.2100746, PMID: 35551702 PMC9101966

[B24] Obeagu E. I. (2025). Monocytes and parturition: Linking prolonged labor to immune dysregulation. Medicine 104, e42351. doi: 10.1097/MD.0000000000042351, PMID: 40295229 PMC12040055

[B25] Peluso M. J. Deitchman A. N. Torres L. Iyer N. S. Munter S. E. Nixon C. C. . (2021). Long-term SARS-CoV-2-specific immune and inflammatory responses in individuals recovering from COVID-19 with and without post-acute symptoms. Cell Rep. 36, 109518. doi: 10.1016/j.celrep.2021.109518, PMID: 34358460 PMC8342976

[B26] Romanyuk V. Raichel L. Sergienko R. Sheiner E. (2011). Pneumonia during pregnancy: radiological characteristics, predisposing factors and pregnancy outcomes. J. Matern Fetal Neonatal Med. 24, 113–117. doi: 10.3109/14767051003678275, PMID: 20476873

[B27] Ryan F. J. Hope C. M. Masavuli M. G. Lynn M. A. Mekonnen Z. A. Yeow A. E. L. . (2022). Long-term perturbation of the peripheral immune system months after SARS-CoV-2 infection. BMC Med. 20, 26. doi: 10.1186/s12916-021-02228-6, PMID: 35027067 PMC8758383

[B28] Sullivan G. M. Feinn R. (2012). Using effect size—or why the P value is not enough. J. Graduate Med. Educ. 4, 279–282. doi: 10.4300/JGME-D-12-00156.1, PMID: 23997866 PMC3444174

[B29] Tang P. Wang J. Song Y. (2018). Characteristics and pregnancy outcomes of patients with severe pneumonia complicating pregnancy: a retrospective study of 12 cases and a literature review. BMC Pregnancy Childbirth 18, 434. doi: 10.1186/s12884-018-2070-0, PMID: 30390683 PMC6215647

[B30] Wu Q. Pan X. Han D. Ma Z. Zhang H. (2024). New Insights into the Epidemiological Characteristics of Mycoplasma pneumoniae Infection before and after the COVID-19 Pandemic. Microorganisms 12, 2019. doi: 10.3390/microorganisms12102019, PMID: 39458327 PMC11509874

[B31] Yang S. Lu S. Guo Y. Luan W. Liu J. Wang L. (2024). A comparative study of general and severe mycoplasma pneumoniae pneumonia in children. BMC Infect. Dis. 24, 449. doi: 10.1186/s12879-024-09340-x, PMID: 38671341 PMC11046970

[B32] Zhang X.-B. He W. Gui Y.-H. Lu Q. Yin Y. Zhang J.-H. . (2024). Current Mycoplasma pneumoniae epidemic among children in Shanghai: unusual pneumonia caused by usual pathogen. World J. Pediatr. 20, 5–10. doi: 10.1007/s12519-023-00793-9, PMID: 38231466

[B33] Zhao Y. Ma G. Yang X. (2019). HDAC5 promotes Mycoplasma pneumoniae-induced inflammation in macrophages through NF-κB activation. Life Sci. 221, 13–19. doi: 10.1016/j.lfs.2019.02.004, PMID: 30738045

