# Supplementary material for: Association between Mycoplasma pneumoniae infection and adverse pregnancy outcome: a propensity score weighting study
Source: Front Cell Infect Microbiol. 2025 Dec 24;15:1663272. doi: 10.3389/fcimb.2025.1663272 (PMC12775152; doi:10.3389/fcimb.2025.1663272)

**Supplementary Materials**

**Table S1.** **Variable Encoding and Proportion of Missing Data.**

| **Variable (Encoding)** | **Control (n=137)** | ***M. pneumoniae* (n=49)** |
| --- | --- | --- |
| **Baseline** |  |  |
| Age (Continuous) | 0/137 (0.0%) | 0/49 (0.0%) |
| BMI (Continuous) | 4/137 (2.9%) | 0/49 (0.0%) |
| Gravidity (3 category: 1,2,3) | 0/137 (0.0%) | 0/49 (0.0%) |
| Abortion (Binary: 0,1) | 0/137 (0.0%) | 0/49 (0.0%) |
| History of chronic diseases (Binary: 0,1) | 0/137 (0.0%) | 0/49 (0.0%) |
| **Clinical characteristics of *M. pneumoniae* infection** |  |  |
| Fever (Binary: 0,1) | 137/137 (100.0%) | 0/49 (0.0%) |
| Cough (Binary: 0,1) | 137/137 (100.0%) | 0/49 (0.0%) |
| Expectoration (Binary: 0,1) | 137/137 (100.0%) | 0/49 (0.0%) |
| Pharyngodynia (Binary: 0,1) | 137/137 (100.0%) | 0/49 (0.0%) |
| Nasal congestion (Binary: 0,1) | 137/137 (100.0%) | 0/49 (0.0%) |
| Rhinorrhea (Binary: 0,1) | 137/137 (100.0%) | 0/49 (0.0%) |
| Headache (Binary: 0,1) | 137/137 (100.0%) | 0/49 (0.0%) |
| Asthenia (Binary: 0,1) | 137/137 (100.0%) | 0/49 (0.0%) |
| Dyspnea (Binary: 0,1) | 137/137 (100.0%) | 0/49 (0.0%) |
| Number of symptoms (Continuous) | 137/137 (100.0%) | 0/49 (0.0%) |
| Duration of symptoms (Continuous) | 137/137 (100.0%) | 0/49 (0.0%) |
| Duration of hospitalization (Continuous) | 137/137 (100.0%) | 0/49 (0.0%) |
| Any complication of *M. pneumoniae* infection (Binary: 0,1) | 137/137 (100.0%) | 0/49 (0.0%) |
| WBC (Continuous) | 137/137 (100.0%) | 0/49 (0.0%) |
| LYM (Continuous) | 137/137 (100.0%) | 3/49 (6.1%) |
| PLT (Continuous) | 137/137 (100.0%) | 0/49 (0.0%) |
| Hb (Continuous) | 137/137 (100.0%) | 0/49 (0.0%) |
| CRP (Continuous) | 137/137 (100.0%) | 2/49 (4.1%) |
| PCT (Continuous) | 137/137 (100.0%) | 7/49 (14.3%) |
| IL-6 (Continuous) | 137/137 (100.0%) | 21/49 (42.9%) |
| ALT (Continuous) | 137/137 (100.0%) | 4/49 (8.2%) |
| AST (Continuous) | 137/137 (100.0%) | 4/49 (8.2%) |
| sCr (Continuous) | 137/137 (100.0%) | 4/49 (8.2%) |

**Table S1. (continued)**

| **Variable (Encoding)** | **Control (n=137)** | ***M. pneumoniae* (n=49)** |
| --- | --- | --- |
| **Adverse** **maternal outcomes** |  |  |
| Adverse maternal events (Binary: 0,1) | 0/137 (0.0%) | 0/49 (0.0%) |
| Cesarean section (Binary: 0,1) | 0/137 (0.0%) | 0/49 (0.0%) |
| PPH (Binary: 0,1) | 0/137 (0.0%) | 0/49 (0.0%) |
| Polyhydramnios or oligohydramnios (Binary: 0,1) | 0/137 (0.0%) | 0/49 (0.0%) |
| Amniotic fluid contamination (Binary: 0,1) | 0/137 (0.0%) | 0/49 (0.0%) |
| Placental abruption (Binary: 0,1) | 0/137 (0.0%) | 0/49 (0.0%) |
| PROM (Binary: 0,1) | 0/137 (0.0%) | 0/49 (0.0%) |
| **Adverse** **neonatal outcomes** |  |  |
| Adverse neonatal events (Binary: 0,1) | 0/137 (0.0%) | 0/49 (0.0%) |
| Fetal HRV (Binary: 0,1) | 0/137 (0.0%) | 0/49 (0.0%) |
| Preterm infant (Binary: 0,1) | 0/137 (0.0%) | 0/49 (0.0%) |
| Fetal distress (Binary: 0,1) | 0/137 (0.0%) | 0/49 (0.0%) |
| Other neonatal infection (Binary: 0,1) | 0/137 (0.0%) | 0/49 (0.0%) |
| Neonatal length (Continuous) | 0/137 (0.0%) | 0/49 (0.0%) |
| Neonatal weight (Continuous) | 0/137 (0.0%) | 0/49 (0.0%) |
| Neonatal head circumference (Continuous) | 0/137 (0.0%) | 0/49 (0.0%) |
| One-minute Apgar score (Continuous) | 0/137 (0.0%) | 0/49 (0.0%) |

Abbreviations: ALT, alanine aminotransferase; AST, aspartate transaminase; BMI, body mass index; CRP, C-reactive protein; CS, cesarean section; Hb, hemoglobin; HRV, heart rate variability; IL-6, Interleukin-6; LYM, lymphocyte; PCT, procalcitonin; PLT, platelets; PPH, postpartum hemorrhage; PROM, premature rupture of membranes; SCr, serum creatinine; WBC, white blood cell.

**Table S2. R Packages and Versions Used in the Statistical Analyses.**

| **Package** | **Version** | **Description** |
| --- | --- | --- |
| R Core | 4.3.1 | R Statistical Computing Environment |
| brglm2 | 0.9.2 | Bias Reduction in Generalized Linear Models |
| dplyr | 1.1.1 | A Grammar of Data Manipulation |
| forestploter | 1.1.1 | Create Flexible Forest Plot |
| ggplot2 | 3.4.3 | Create Elegant Data Visualisations Using the Grammar of Graphics |
| MatchThem | 1.1.0 | Matching and Weighting Multiply Imputed Datasets |
| mice | 3.17.0 | Multivariate Imputation by Chained Equations |
| readxl | 1.4.3 | Read Excel Files |
| rio | 0.5.30 | A Swiss-Army Knife for Data I/O |
| tableone | 0.13.2 | Create 'Table 1' to Describe Baseline Characteristics with or without Propensity Score Weights |
| WeightIt | 0.14.2 | Weighting for Covariate Balance in Observational Studies |

**Table S3. Baseline Characteristics of Participants before and after Propensity Score Matching.**

|  | **Unadjusted** | | |  | **PSM** | | |
| --- | --- | --- | --- | --- | --- | --- | --- |
| **Baseline** | **Control**  **(n=137)** | ***M. pneumoniae***  **(n=49)** | **ASMD** |  | **Control**  **(n=48)** | ***M. pneumoniae***  **(n=48)** | **ASMD** |
| Age, Mean ± SD | 30.42±4.37 | 29.18±3.79 | 0.301 |  | 29.38 (4.37) | 29.27 (3.78) | 0.025 |
| BMI, Mean ± SD | 26.5±3.74 | 25.65±3.51 | 0.238 |  | 25.87 (3.11) | 25.71 (3.52) | 0.049 |
| Gravidity, n (%) |  |  | 0.198 |  |  |  | 0.092 |
| 1 | 58(42.3) | 22(44.9) |  |  | 24 (50.0) | 22 (45.8) |  |
| 2 | 46(33.6) | 19(38.8) |  |  | 16 (33.3) | 18 (37.5) |  |
| 3 | 33(24.1) | 8(16.3) |  |  | 8 (16.7) | 8 (16.7) |  |
| History of abortion, n (%) | 40(29.2) | 15(30.6) | 0.031 |  | 14 (29.2) | 15 (31.2) | 0.045 |
| History of chronic diseases, n (%) | 22(16.1) | 17(34.7) | 0.438 |  | 16 (33.3) | 16 (33.3) | <0.001 |

An ASMD of < 0.10 was defined as an acceptable covariate balance. Abbreviations: ASMD, absolute standardized mean difference; BMI, body mass index; PSM, propensity score matching; SD, standard deviation.

**Table S4. Effects of *M. pneumoniae* Infection on Pregnant Women before and after Propensity Score Matching.**

| **Outcomes** | **Unadjusted** | | | |  | **PSM** | | | |
| --- | --- | --- | --- | --- | --- | --- | --- | --- | --- |
|  | **Control**  **(n=137)** | ***M. pneumoniae***  **(n=49)** | **OR/MD** | ***p*** |  | **Control**  **(n=48)** | ***M. pneumoniae***  **(n=48)** | **OR/MD** | ***p*** |
| Maternal adverse event, n (%) # | 92 (67.15) | 34 (69.39) | 1.11 (0.55-2.25) | 0.774 |  | 28 (58.33) | 33 (68.75) | 1.59 (0.68-3.74) | 0.286 |
| CS, n (%) | 60 (43.80) | 28 (57.14) | 1.71 (0.88-3.32) | 0.112 |  | 20 (41.67) | 27 (56.25) | 2.03 (0.86-4.77) | 0.104 |
| PPH, n (%) ^†^ | 1 (0.73) | 0 (0.00) | 0.00 (0.00-Inf) | 0.997 |  | 0 (0.00) | 0 (0.00) | 1.00 (0.02-56.54) | 1.000 |
| Polyhydramnios or oligohydramnios, n (%) | 16 (11.68) | 11 (22.45) | 2.19 (0.93-5.15) | 0.072 |  | 9 (18.75) | 11 (22.92) | 1.36 (0.46-4.00) | 0.570 |
| Amniotic fluid contamination, n (%) | 16 (11.68) | 6 (12.24) | 1.06 (0.39-2.89) | 0.916 |  | 4 (8.33) | 6 (12.50) | 1.53 (0.39-6.03) | 0.539 |
| Placental abruption, n (%) ^†^ | 1 (0.73) | 1 (2.04) | 2.83 (0.17-47.05) | 0.466 |  | 0 (0.00) | 1 (2.08) | 3.06 (0.11-83.36) | 0.502 |
| PROM, n (%) | 34 (24.82) | 10 (20.41) | 0.78 (0.35-1.73) | 0.535 |  | 9 (18.75) | 10 (20.83) | 1.21 (0.39-3.76) | 0.737 |
| Neonatal adverse event, n (%) # | 65 (47.45) | 24 (48.98) | 1.06 (0.55-2.05) | 0.854 |  | 21 (43.75) | 24 (50.00) | 1.14 (0.49-2.67) | 0.754 |
| Fetal HRV, n (%) | 26 (18.98) | 14 (28.57) | 1.71 (0.80-3.64) | 0.165 |  | 9 (18.75) | 14 (29.17) | 1.70 (0.60-4.79) | 0.311 |
| Preterm infant, n (%) | 17 (12.41) | 8 (16.33) | 1.38 (0.55-3.45) | 0.492 |  | 5 (10.42) | 7 (14.58) | 1.53 (0.41-5.70) | 0.521 |
| Fetal distress, n (%) | 24 (17.52) | 10 (20.41) | 1.21 (0.53-2.76) | 0.654 |  | 7 (14.58) | 10 (20.83) | 1.15 (0.35-3.75) | 0.808 |
| Other neonatal infection, n (%) | 7 (5.11) | 6 (12.24) | 2.59 (0.82-8.19) | 0.104 |  | 4 (8.33) | 6 (12.50) | 2.17 (0.45-10.49) | 0.327 |
| Neonatal length, cm, Mean ± SD | 49.58±3.01 | 49.31±2.22 | -0.27 (-1.20-0.66) | 0.566 |  | 49.54±2.42 | 49.40±2.15 | -0.06 (-1.02-0.90) | 0.902 |
| Neonatal weight, cm, Mean ± SD | 3.05±0.53 | 3.01±0.52 | -0.05 (-0.22-0.12) | 0.586 |  | 3.08±0.47 | 3.02±0.51 | -0.04 (-0.25-0.17) | 0.718 |
| Neonatal head circumference, cm, Mean ± SD | 33.04±1.93 | 32.96±1.55 | -0.08 (-0.68-0.53) | 0.800 |  | 33.32±1.79 | 32.97±1.56 | -0.21 (-0.93-0.51) | 0.569 |
| One-minute Apgar score, Mean ± SD | 8.93±0.30 | 8.90±0.51 | -0.04 (-0.16-0.08) | 0.554 |  | 8.98±0.14 | 8.90±0.52 | -0.07 (-0.23-0.10) | 0.416 |

#The primary outcome. ^†^Firth’s penalized logistic regression to correct sparse data bias.

Abbreviations: CS, cesarean section; HRV, heart rate variability; MD, mean difference; OR, odd ratio; PPH, postpartum hemorrhage; PROM, premature rupture of membranes; PSM, propensity score matching; SD, standard deviation.

**Figure S1. Subgroup Analysis of PPH with PSW.** ^†^Firth’s penalized logistic regression to correct sparse data bias. Abbreviations: OR, odd ratio; PPH, postpartum hemorrhage; PSW, propensity score weighting.


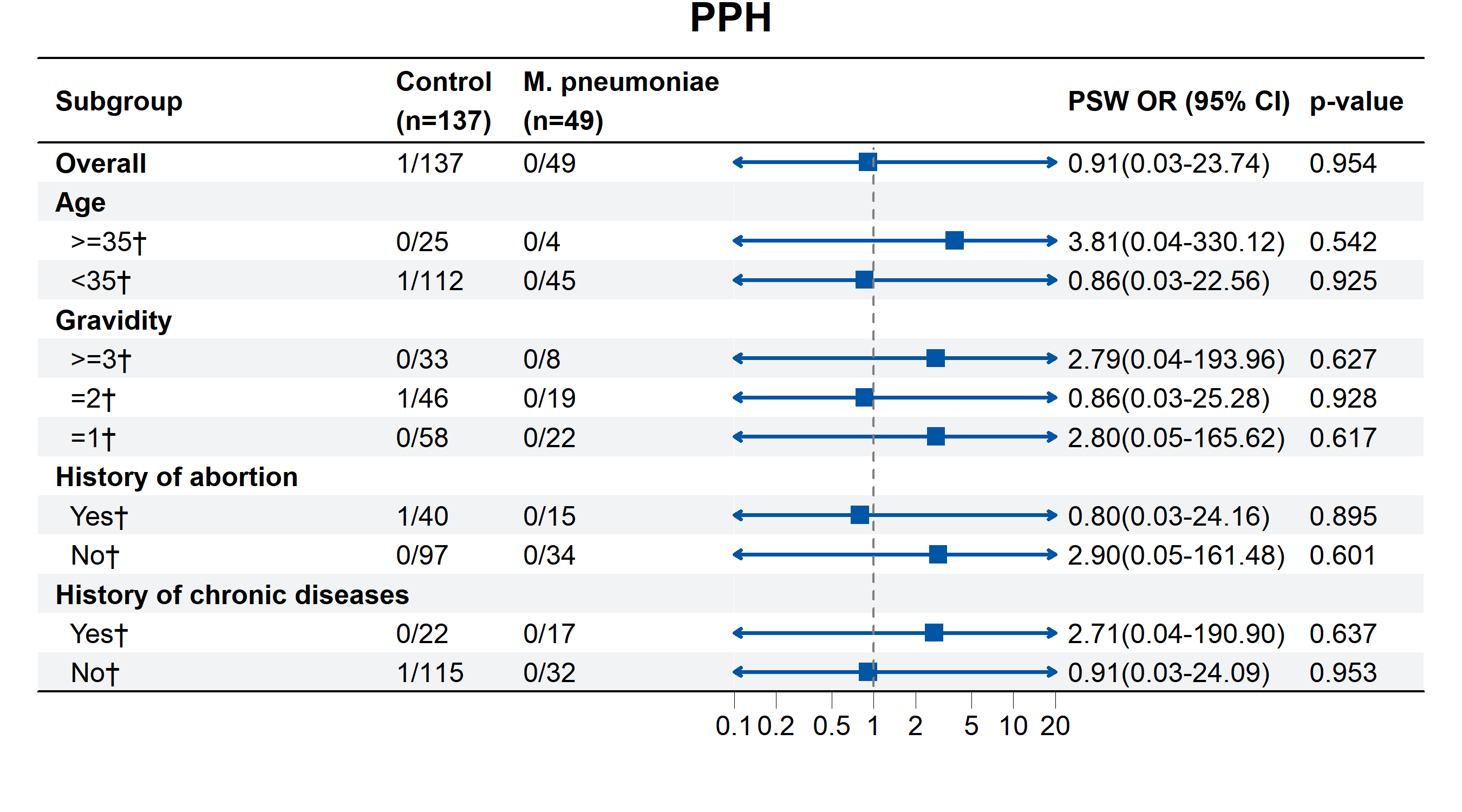


**Figure S2. Subgroup Analysis of Polyhydramnios or Oligohydramnios with PSW.** Abbreviations: OR, odd ratio; PSW, propensity score weighting.


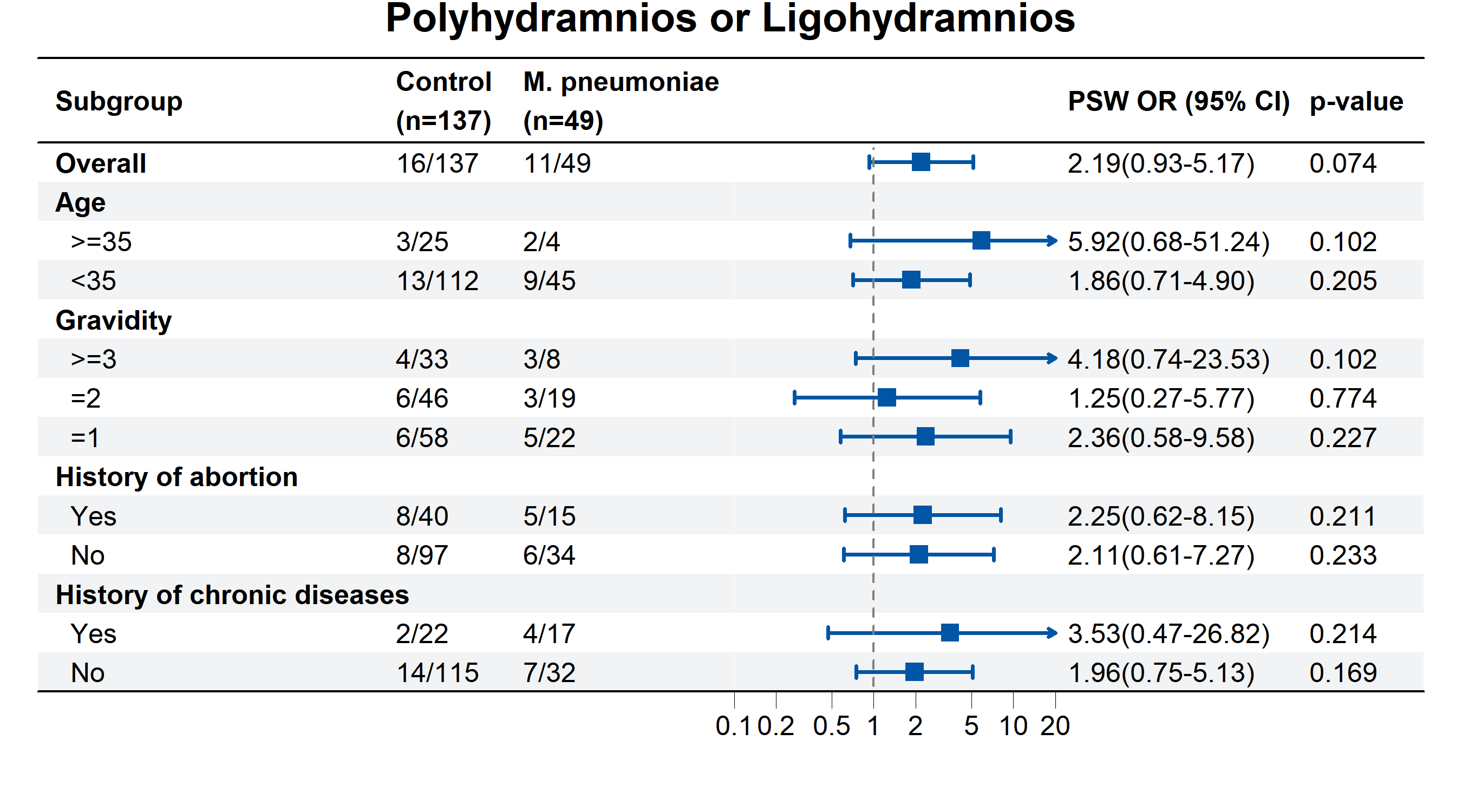


**Figure S3. Subgroup Analysis of Amniotic Fluid Contamination with PSW.** ^†^Firth’s penalized logistic regression to correct sparse data bias. Abbreviations: OR, odd ratio; PSW, propensity score weighting.


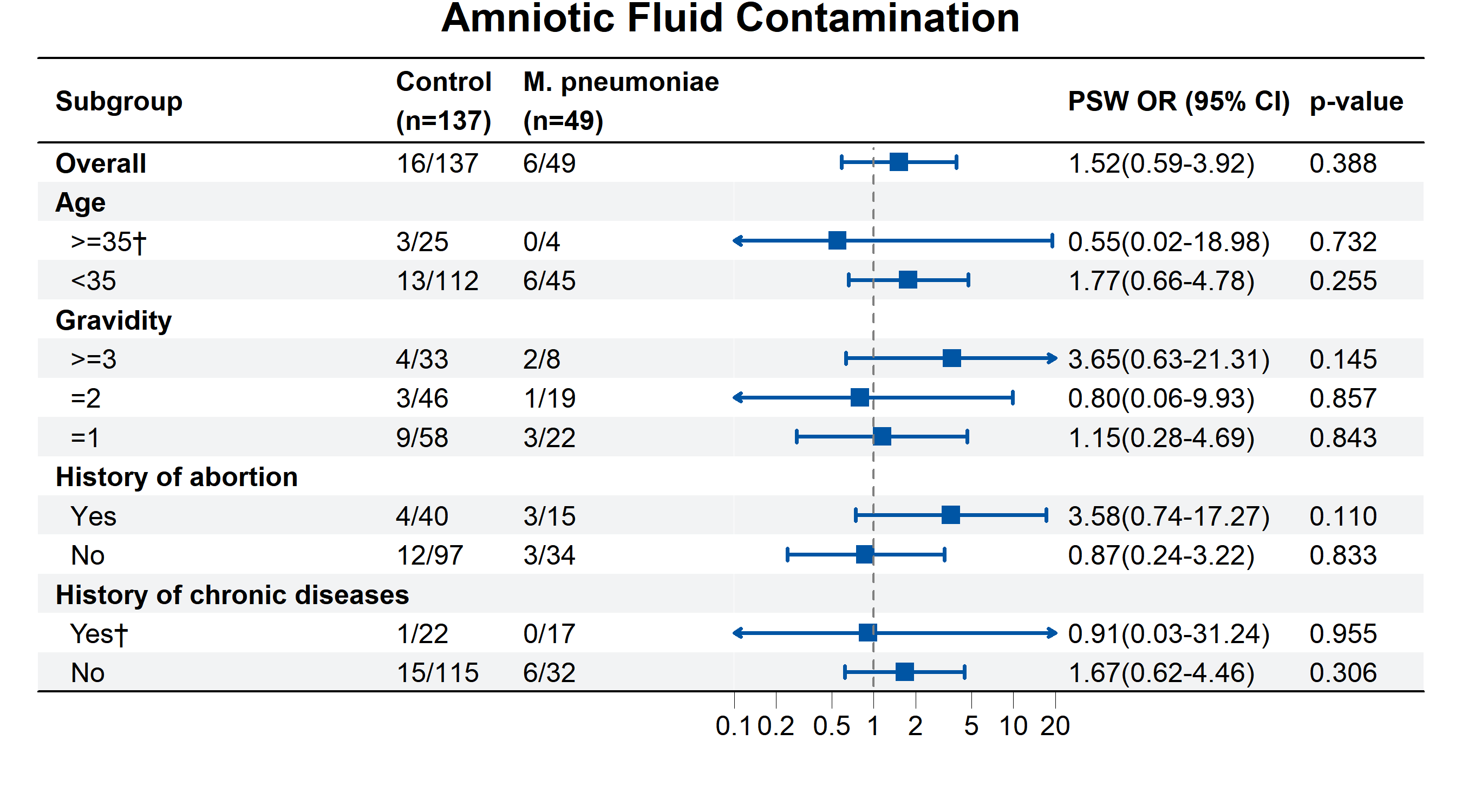


**Figure S4. Subgroup Analysis of Placental Abruption with PSW.** ^†^Firth’s penalized logistic regression to correct sparse data bias. Abbreviations: MD, mean difference; OR, odd ratio; PSW, propensity score weighting;


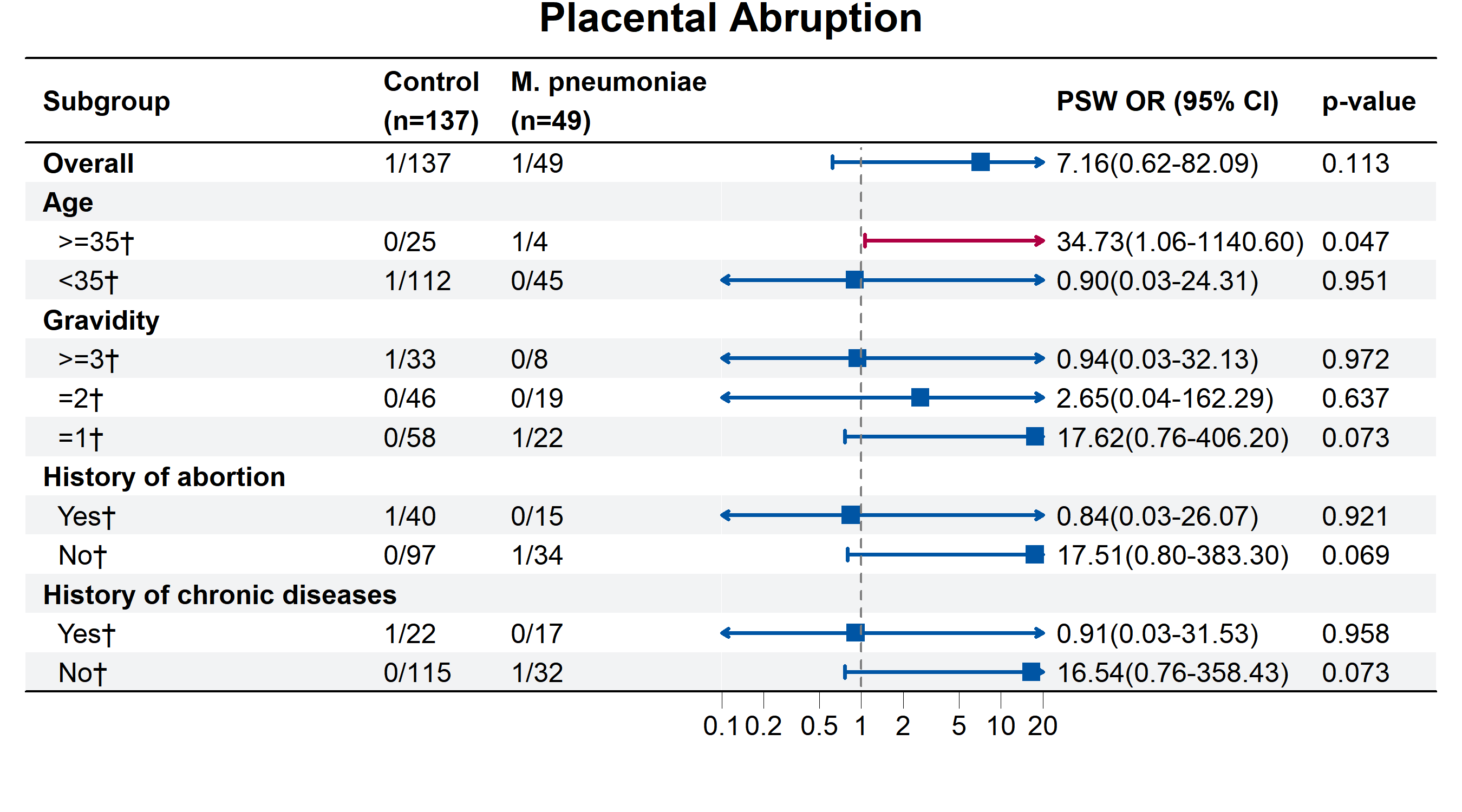


**Figure S5. Subgroup Analysis of PROM with PSW.** ^†^Firth’s penalized logistic regression to correct sparse data bias. Abbreviations: OR, odd ratio PROM, premature rupture of membranes; PSW, propensity score weighting.


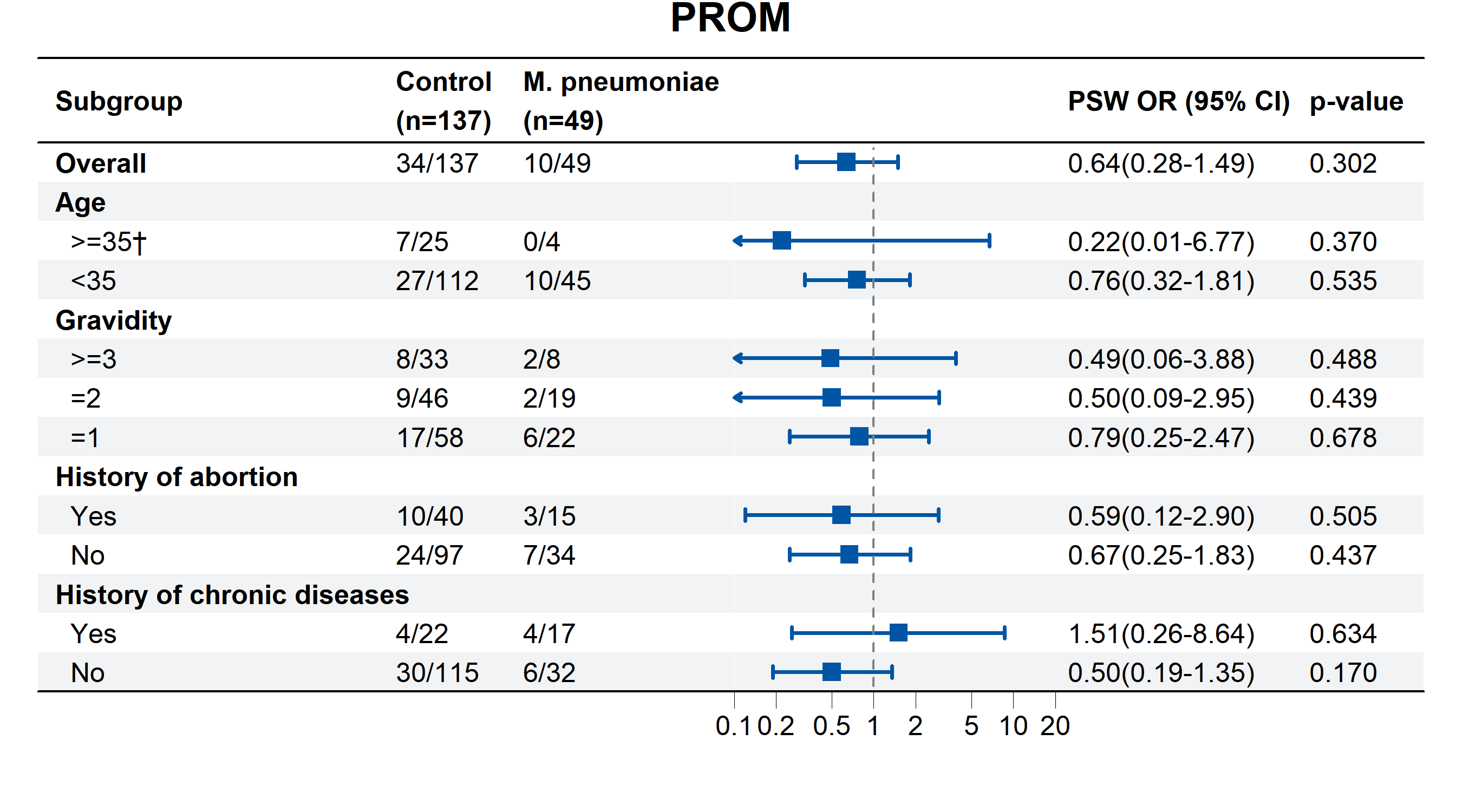


**Figure S6. Subgroup Analysis of Adverse Neonatal Events with PSW.** ^†^Firth’s penalized logistic regression to correct sparse data bias. Abbreviations: OR, odd ratio; PSW, propensity score weighting.


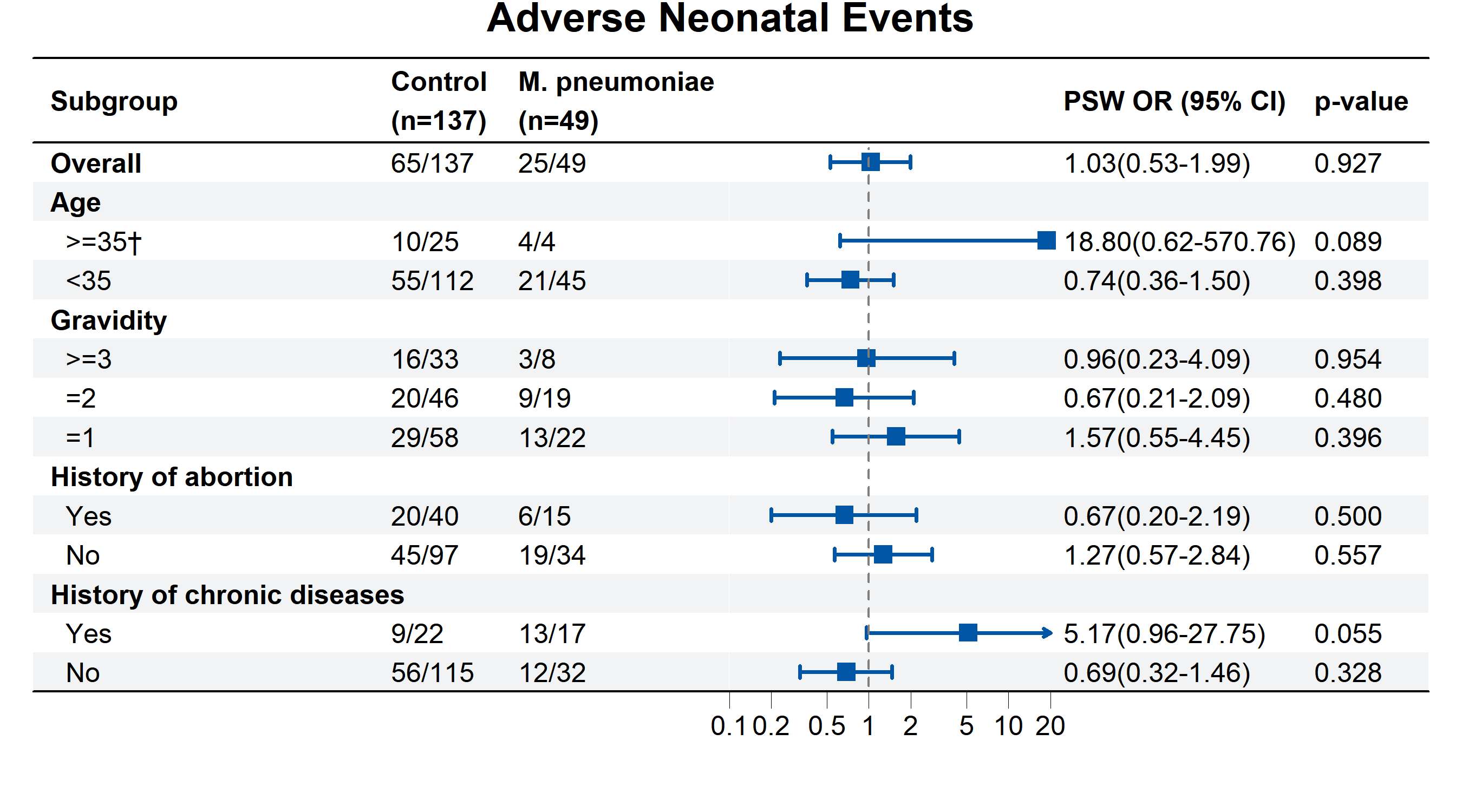


**Figure S7. Subgroup Analysis of Fetal HRV with PSW.** Abbreviations: HRV, heart rate variability; OR, odd ratio; PSW, propensity score weighting.


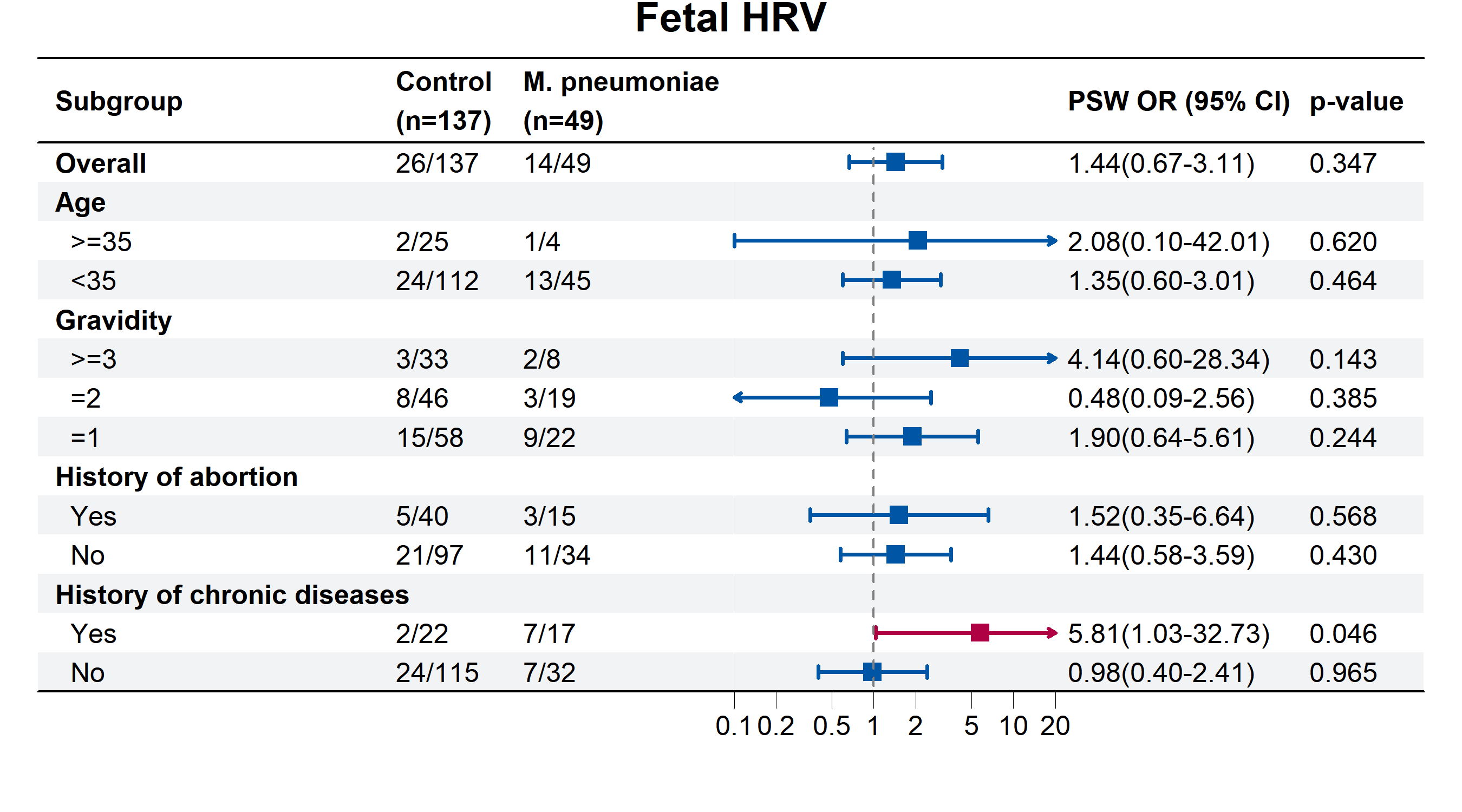


**Figure S8. Subgroup Analysis of Other Neonatal Infection with PSW.** ^†^Firth’s penalized logistic regression to correct sparse data bias. Abbreviations: OR, odd ratio; PSW, propensity score weighting.


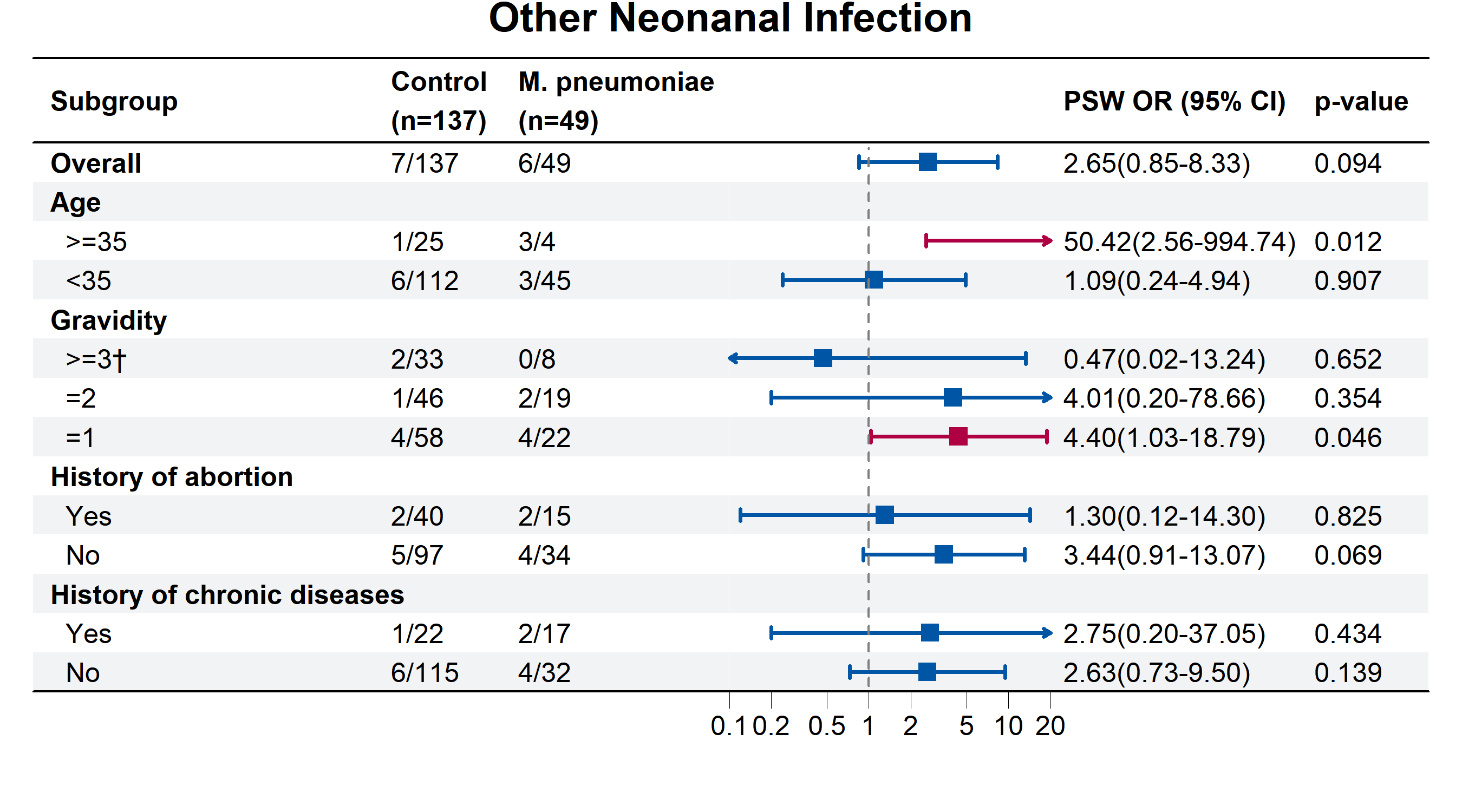


**Figure S9. Subgroup Analysis of Neonatal Length with PSW.** Abbreviations: PSW, propensity score weighting.


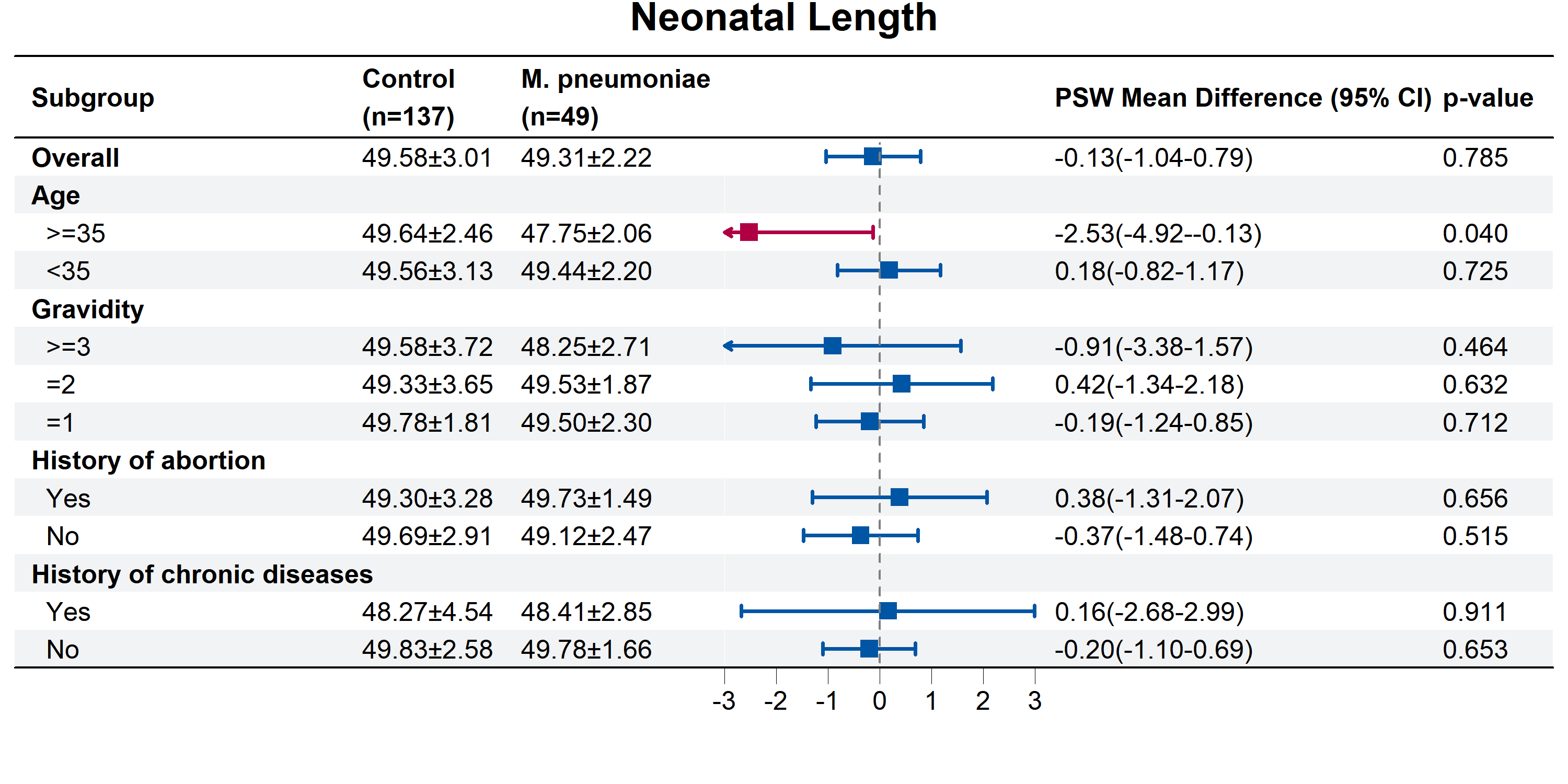


**Figure S10. Subgroup Analysis of Neonatal Weight with PSW.** Abbreviations: PSW, propensity score weighting.


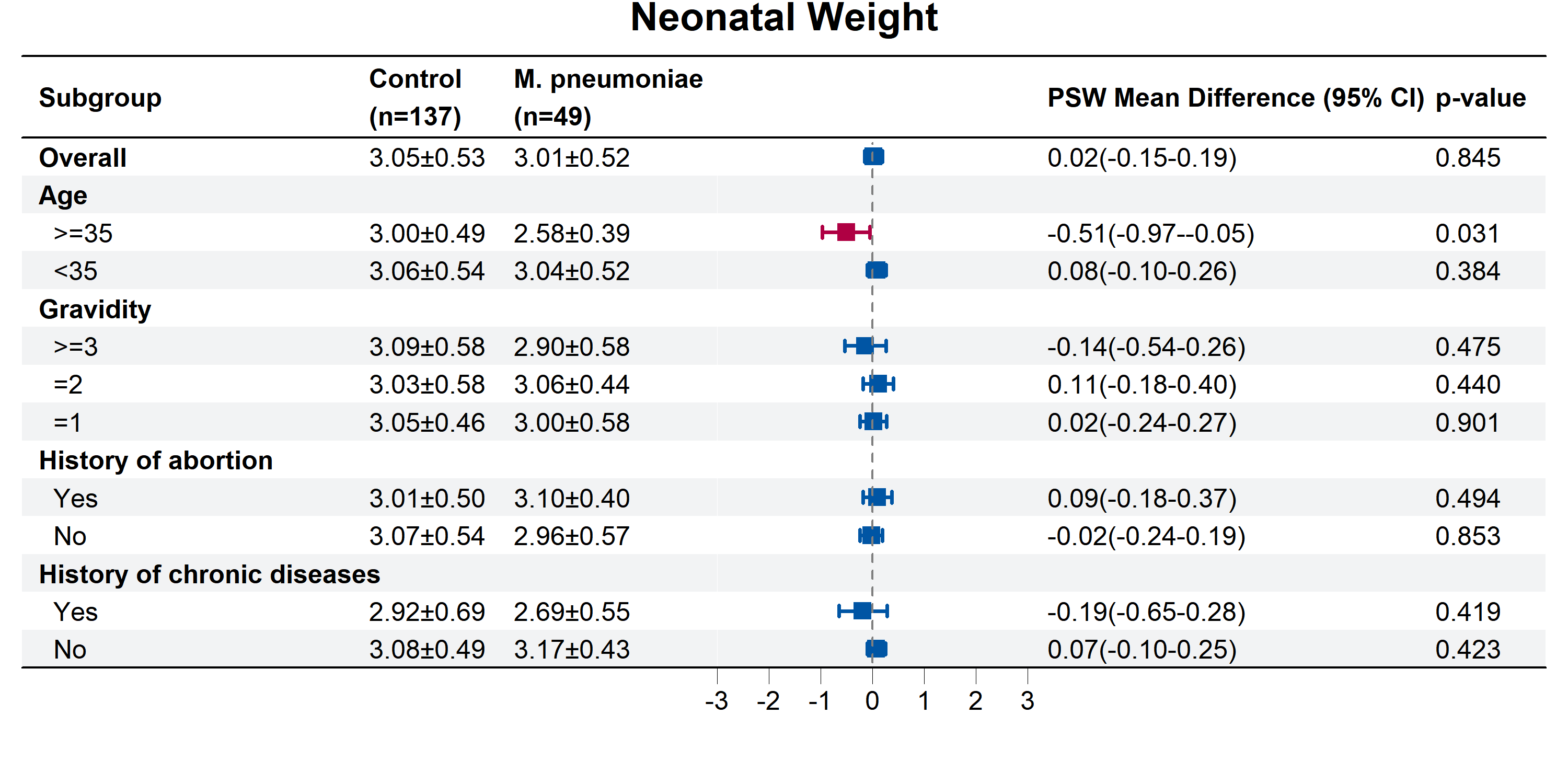


**Figure S11. Subgroup Analysis of Neonatal Head Circumference with PSW.** Abbreviations: PSW, propensity score weighting.


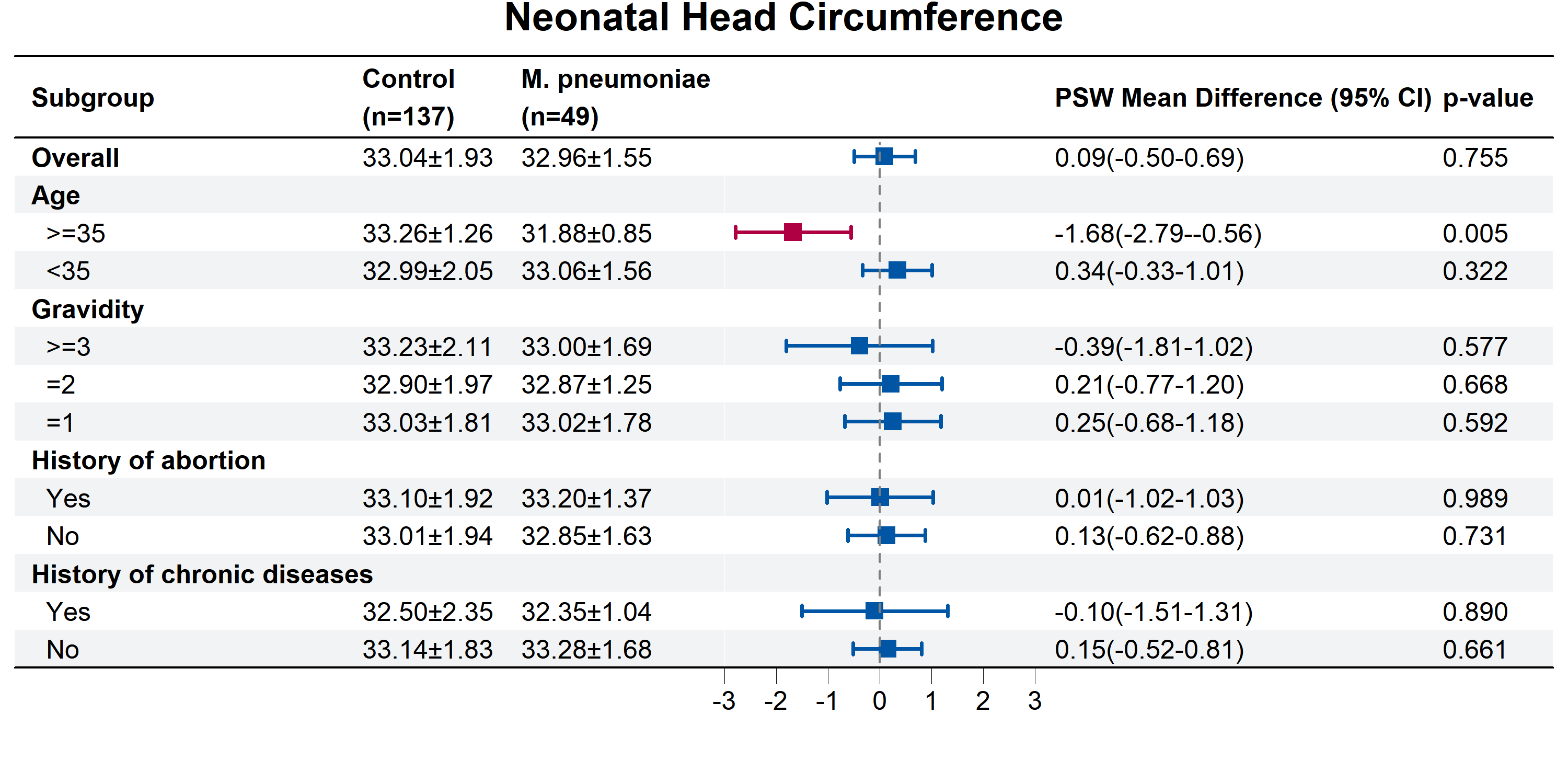


**Figure S12. Subgroup Analysis of One-Minute Apgar Score with PSW.** Abbreviations: PSW, propensity score weighting.


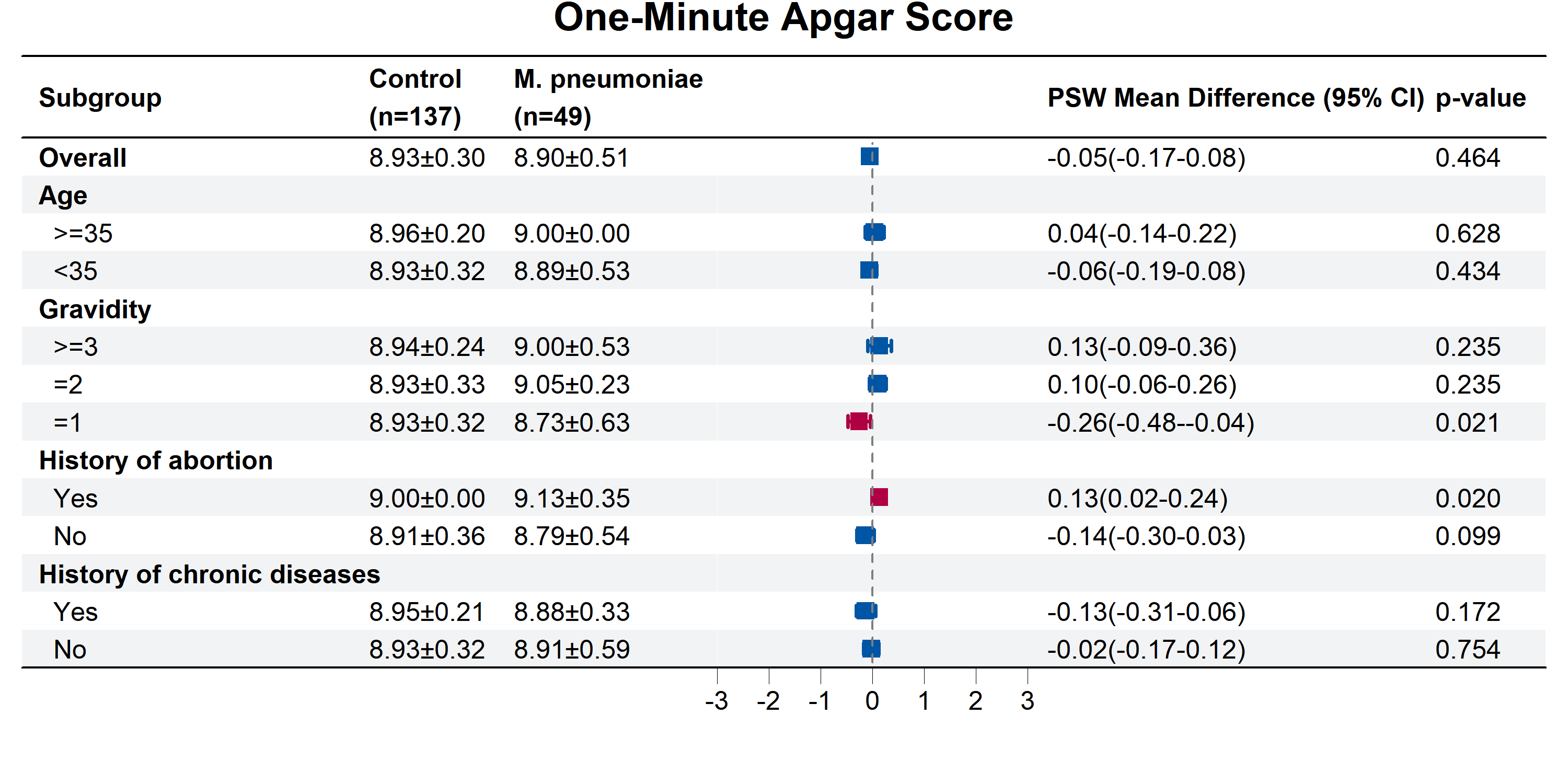


**Figure S13. Post-hoc power analysis.** P0 denotes the baseline risk. N=186 participants, with 49 in the exposure group (26.3%) and 137 in the control group (73.6%).


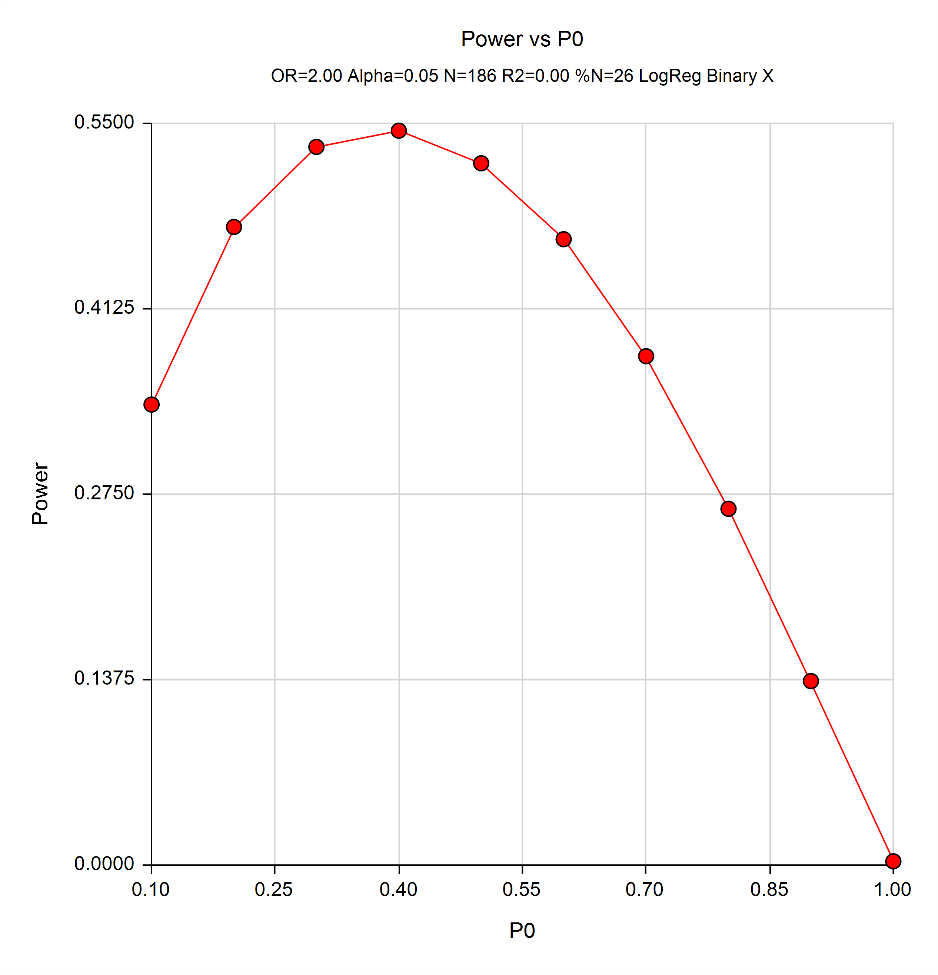

Supplement: Supplementary file 1 [file DataSheet1.docx]
